# Antiviral Nanobiologic Therapy Remodulates Innate Immune Responses to Highly Pathogenic Coronavirus

**DOI:** 10.1002/advs.202207249

**Published:** 2023-04-25

**Authors:** Xuan Liu, Lunzhi Yuan, Jijing Chen, Yali Zhang, Peiwen Chen, Ming Zhou, Jiaxuan Xie, Jian Ma, Jianzhong Zhang, Kun Wu, Qiyi Tang, Quan Yuan, Huachen Zhu, Tong Cheng, Yi Guan, Gang Liu, Ningshao Xia

**Affiliations:** ^1^ State Key Laboratory of Molecular Vaccinology and Molecular Diagnostics National Institute of Diagnostics and Vaccine Development in Infectious Diseases Center for Molecular Imaging and Translational Medicine School of Public Health & School of Life Sciences Xiamen University Xiamen 361102 China; ^2^ State Key Laboratory of Emerging Infectious Diseases School of Public Health Li Ka Shing Faculty of Medicine The University of Hong Kong Hong Kong SAR 999077 China; ^3^ Guangdong‐Hong Kong Joint Laboratory of Emerging Infectious Diseases Joint Laboratory for International Collaboration in Virology and Emerging Infectious Diseases Joint Institute of Virology (STU/HKU) Shantou University Shantou 515063 China; ^4^ Department of Microbiology Howard University College of Medicine Washington DC 20059 USA

**Keywords:** ARDS, biomimetic nanocarrier, cell membrane vesicles, endogenous type I interferon, highly pathogenic coronavirus, imbalanced innate immune responses

## Abstract

Highly pathogenic coronavirus (CoV) infection induces a defective innate antiviral immune response coupled with the dysregulated release of proinflammatory cytokines and finally results in acute respiratory distress syndrome (ARDS). A timely and appropriate triggering of innate antiviral response is crucial to inhibit viral replication and prevent ARDS. However, current medical countermeasures can rarely meet this urgent demand. Here, an antiviral nanobiologic named CoVR‐MV is developed, which is polymerized of CoVs receptors based on a biomimetic membrane vesicle system. The designed CoVR‐MV interferes with the viral infection by absorbing the viruses with maximized viral spike target interface, and mediates the clearance of the virus through its inherent interaction with macrophages. Furthermore, CoVR‐MV coupled with the virus promotes a swift production and signaling of endogenous type I interferon via deregulating 7‐dehydrocholesterol reductase (DHCR7) inhibition of interferon regulatory factor 3 (IRF3) activation in macrophages. These sequential processes re‐modulate the innate immune responses to the virus, trigger spontaneous innate antiviral defenses, and rescue infected Syrian hamsters from ARDS caused by SARS‐CoV‐2 and all tested variants.

## Graphical abstract

IFN‐I‐dependent endogenous antiviral response is vital to inhibit highly pathogenic coronavirus replication and prevent ARDS. A biomimetic antiviral nanobiologic administered intranasally breaks the general mode of action of direct‐acting antivirals, triggers a swift production and signaling of IFN‐I, and reverses the imbalanced innate immune responses to defend SARS‐CoV‐2 that with high mutation rates and fairly strong immunepathogenicity.

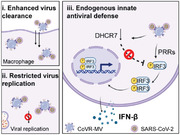

## 1 Introduction

The global expansion of coronavirus disease 2019 (COVID‐19) caused by severe acute respiratory syndrome coronavirus 2 (SARS‐CoV‐2) has emerged as a great public health crisis. Common with previous outbreaks of the two highly pathogenic coronaviruses, including severe acute respiratory syndrome coronavirus (SARS‐CoV) and middle east respiratory syndrome coronavirus (MERS‐CoV), SARS‐CoV‐2 can also lead to acute respiratory distress syndrome (ARDS). The progress of lethal ARDS does not seem to be solely related to robust infection and could involve a defective antiviral immune response coupled with the dysregulated release of proinflammatory cytokines.^[^ ^1^ ^]^ Innate immunity plays a vital role in eliminating viruses through initiating type I interferons (IFN‐I)‐dependent antiviral responses. Upon virus invasion, pathogen‐associated molecular patterns (PAMPs) are recognized by pattern recognition receptors (PRRs), and signaling cascades are induced that result in type I interferons (IFN‐*α*/*β*) production to activate IFN‐I signaling pathways. However, growing evidence has demonstrated that endogenous IFN‐*α*/*β* production and signaling are significantly blunted and counteracted due to multiple strategies that evolved by coronaviruses.^[^ ^2^ ^]^ Consequently, robust viral replication in the lung triggers an excessive inflammatory response, which may become explosive and potentially result in a cytokine storm, diffuse lung tissue injury and ARDS.^[^ ^3^ ^]^ Besides SARS‐CoV‐2, it has been proved that the innate immune responses were dysregulated in both SARS‐CoV and MERS‐CoV infections.^[^ ^4^ ^]^

An in‐depth understanding of the interplay between virus and host innate immunity is crucial to develop therapeutic strategies. Different viral loads led to varied immune responses and severity of disease outcomes,^[^ ^5^ ^]^ which indicates that a decrease in viral load may attenuate its pathogenicity by decreasing the impairment to early host antiviral response and suppressing the process of immunopathogenesis.^[^ ^6^ ^]^ However, the efficiency of approved antiviral agents has been challenged by the high mutation rate and increasing immune escape ability of newly emerged variants. Furthermore, the decrease in viral load in the end stage of COVID‐19 is ineffective in reversing the severe persisting inflammation and diffusing lung tissue injury, especially in critical cases. On the other hand, anti‐inflammatory drugs usually lack the function of viral suppression, which might limit their therapeutic effect and cause side effects. For instance, in previous studies, we found that, as a clinically commonly used anti‐inflammatory drug, dexamethasone decreases SARS‐CoV‐2‐caused lung inflammation and pathogenesis, but promotes viral replication.^[^ ^7^ ^]^ Besides the above drawbacks, these passive treatment agents are inadequate to activate the production of endogenous IFN‐*α*/*β*. Therefore, designing the next‐generation medical countermeasures that combine broad‐spectrum antiviral manner and spontaneous immunomodulation to address the imbalanced innate immunity might establish a new paradigm to defend the evolving SARS‐CoV‐2 variants and possible pandemic of highly pathogenic coronavirus in future.

Biomaterials can be functionalized with antiviral molecular and designed to exhibit high phagocyte avidity, and are thus ideal platforms with which to mediate virus clearance and immunomodulation.^[^ ^8^ ^]^ On the other hand, throughout the evolution of SARS‐CoV‐2, new variants rarely change their receptor‐binding preference but usually exhibit an elevated binding affinity. Inspired by these facts, we hypothesize that size‐specific exogenous mimics of receptors could interfere with the viral infection and remodulate innate immune responses to the virus by interacting with it. Over the past five years, we have developed receptor‐based nanodecoys to combat hepatitis B virus infection,^[^ ^9^ ^]^ and then the receptor‐mediated antiviral effect of nanodecoys was widely demonstrated. Most of these therapeutic agents have focused on neutralizing the virus itself while often ignoring their in vivo behavior, especially the interplay with the immune system. In this study, we designed an antiviral agent named CoVR‐MV, which joint displayed of SARS‐CoV/SARS‐CoV‐2 receptor protein human angiotensin‐converting enzyme 2 (hACE2) and MERS‐CoV receptor protein human dipeptidyl peptidase 4 (hDPP4). The designed CoVR‐MVs broadly absorbed SARS‐CoV, MERS‐CoV, SARS‐CoV‐2, and circulating variants by the maximized viral spike target interface. Intranasally administered CoVR‐MVs dexterously navigate the anatomical of the lungs to enter the alveoli and are taken up by alveolar macrophages. In Syrian hamsters with SARS‐CoV‐2 infection, inhaled CoVR‐MVs interfered with the infection behavior of the virus and re‐modulated innate immune responses to the virus, thus triggering spontaneous innate antiviral defenses and rescuing hamsters from cytokine storm, lung pathogenesis and lethal ARDS. In addition, we revealed that CoVR‐MVs therapy promoted endogenous IFN‐I production via deregulating DHCR7 inhibition of IRF3 in macrophages, which further improves our understanding of how CoVR‐MVs therapy could reverse the imbalanced innate immune responses from pro‐inflammatory to antiviral.

## 2 Results

### 2.1 Imbalanced Innate Immune Responses and Robust Respiratory Infection Are Typical Features of Highly Pathogenic Coronavirus‐Caused ARDS

**Figure 1:**
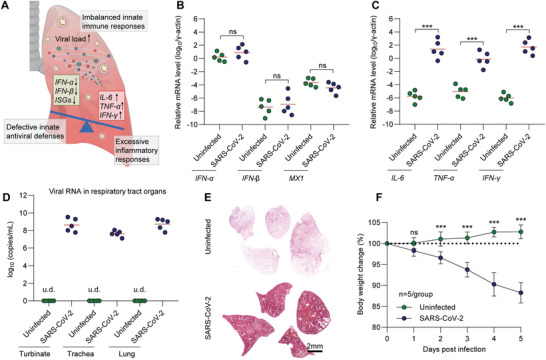
Imbalanced innate immune response and robust respiratory infection are typical features of highly pathogenic coronavirus‐caused ARDS. A) Schematic illustration for the characteristics of imbalanced innate immune responses after highly pathogenic coronavirus infection. Relative mRNA levels of B) *IFN‐* *α*, *IFN‐* *β*, *MX1*, and C) proinflammatory cytokines include *IL‐6, TNF‐α* and *IFN‐γ* in the lung tissues collected at 5 dpi, respectively (*n* = 5). The mRNA levels were standardized to the house‐keeping gene *γ*‐actin. D) Viral RNA levels in turbinate, trachea and lung tissues collected at 5 dpi were measured by RT‐PCR (*n* = 5). The primers of SARS‐CoV‐2 ORF1ab gene were used. E) Representative H&E staining images for lung lobes collected at 5 dpi were shown, Scale bar = 2 mm. F) Body weight changes of the hamsters from 0 to 5 dpi were recorded (*n* = 5). Data were shown as mean ± SD. Statistical analysis for F) was performed using two‐way ANOVA. Other analyses were performed using one‐way ANOVA. *p*‐values <0.05 was considered significant: ***P <0.001, ns indicated no significance to the positive control (*p* > 0.05). u.d. indicated undetectable.

IFN‐I and downstream interferon‐stimulated genes (ISGs) play important roles in host antiviral defense. However, to establish a successful infection of host cells, highly pathogenic coronaviruses have developed multifaceted mechanisms to inhibit IFN‐I induction and signaling.^[^ ^10^ ^]^ It is reported that M, N, ORF6, ORF8, and other proteins of SARS‐CoV‐2 could interfere with innate immune responses,^[^ ^2^, ^11^ ^]^ which accentuated the necessity of combining antivirals and immunomodulators. To characterize the innate immune responses to SARS‐CoV‐2, a sensitive animal model named Syrian hamster with virological, pathological, and immunological characteristics that are similar to human COVID‐19 patients was employed. The hamsters were intranasally inoculated with 1 × 10^4^ plaque‐forming units (PFU) of prototype SARS‐CoV‐2 as previously described.^[^ ^12^ ^]^ The hamsters without infection were set as a control group. Five days after infection (5 dpi), all hamsters were euthanized for immunological and virological examinations. The mRNA levels of antiviral genes *IFN‐α*, *IFN‐β*, and canonical ISG myxovirus‐resistance protein‐1 (*MX1*) (**Figure** **1**B) in lung tissues were not significantly increased at 5 dpi. Whereas, SARS‐CoV‐2 induced a great inflammatory signature, as exemplified by high mRNA levels of typical proinflammatory cytokines like *IL‐6*, *TNF‐α*, and *IFN‐γ* (Figure 1C), consistent with what has been observed in people with COVID‐19.^[^ ^13^ ^]^ Next, we analyzed viral replication in respiratory tract organs, including turbinate, trachea, and lung, by RT‐PCR that amplifies SARS‐CoV‐2 open reading frame 1ab (ORF1ab) to detect viral RNA load in the homogenized tissues collected at 5 dpi. Due to the inadequate antiviral immune responses, a high viral RNA load in respiratory tract organs, including turbinate, trachea, and lung, was detected (Figure 1D). The diffuse lung pathological changes (Figure 1E) and 11.8±2.5% of body weight loss (Figure 1F) at 5 dpi suggested a critical disease outcome of ARDS. Overall, the results of our animal experiments and clinical reports indicated that attenuated innate antiviral defenses coupled with excessive inflammatory cytokine production, and robust viral infections are the driving features of SARS‐CoV‐2‐caused ARDS (Figure 1A). A timely and appropriate innate antiviral response is crucial to inhibit viral replication and prevent the deterioration of ARDS. However, current medical countermeasures can rarely meet this urgent demand. For example, we have demonstrated that the commonly used anti‐inflammation drug dexamethasone can ameliorate severe pneumonia but has no antiviral effect in SARS‐CoV‐2 infected Syrian hamsters.^[^ ^7^ ^]^ Moreover, early intervention of anti‐inflammation drugs might interfere with endogenous antiviral responses by non‐specific immunosuppression. Taken together, these results prompted us to design a new antiviral agent to address the robust respiratory infection and assist in the production of endogenous IFN‐*α*/*β*.

### 2.2 Preparation and Characterization of CoVR‐MVs

**Figure 2:**
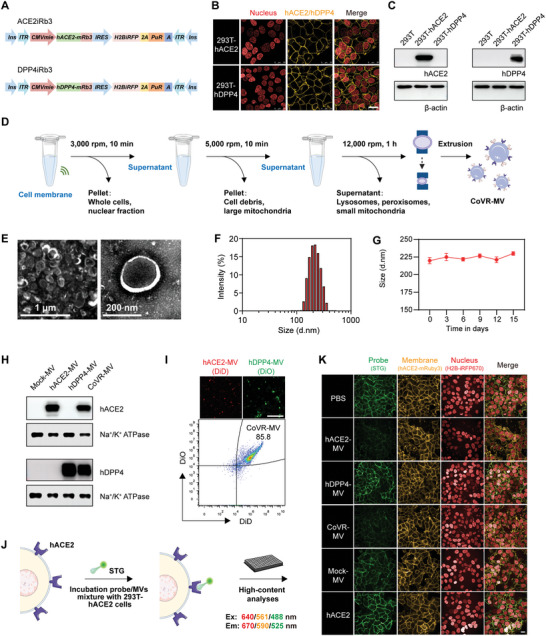
Preparation and characterization of CoVR‐MVs. A) Schematics of the constructs of ACE2iRb3 and DPP4iRb3 for generations of ACE2‐ and hDPP4‐overexpressing cell lines. B) Fluorescence confocal images of 293T‐hACE2‐ and 293T‐hDPP4 cells (Scale bar, 25 µm). C) Western blot analyses of expressions of hACE2 and hDPP4 in 293T‐hACE2 and 293T‐hDPP4 cells. D) Schematic illustration for the generation of CoVR‐MVs. E) Representative transmission electron microscopy image (scale bar, left, 1 µm; right, 200 nm), and F) histogram of the particle size distribution of CoVR‐MVs. G) DLS stability assay demonstrated that CoVR‐MVs size remains stable for at least 15 d (*n* = 3). Data were shown as mean ± SD. H) Western blot analyses of hACE2 and hDPP4 in Mock‐MVs, hACE2‐MVs, hDPP4‐MVs and CoVR‐MVs. I) Fluorescence confocal images of DiD‐hACE2‐MVs and DiO‐hDPP4‐MVs (Scale bar, 10 µm). The fluorescent signal of CoVR‐MVs was detected by flow cytometry. The hACE2‐MVs and hDPP4‐MVs were respectively labeled with DiD and DiO fluorescence dyes before fusion. J) Measurement for the inhibition efficiency of hACE2‐MVs, hDPP4‐MVs, CoVR‐MVs, Mock‐MVs, and soluble hACE2 in a cell‐based SARS‐CoV‐2 spike function blocking test by using fluorescent STG probe and high‐content imaging assay. K) Confocal images of STG, cell membrane and nucleus in 293T‐hACE2 cells at 1 h post STG co‐incubation with each MVs and hACE2, respectively (Scale bar, 20 µm).

The high mutation of highly pathogenic coronaviruses, especially SARS‐CoV‐2, challenges drug development and vaccine efficacy. Inspired by the canon of virus‐receptor binding, we anticipated that the biomimetic vesicle derived from CoVs receptors overexpressing cytomembrane might be a potent blocker for CoVs through multivalent receptor‐pathogen interactions. We have previously shown that, compared to soluble receptors, membrane‐derived vesicles (MVs) are straightforward to be bestowed the natural properties of the source cytomembrane, including maximized virus bio‐interfacing capabilities and conformational equilibria of receptors.^[^ ^9^, ^14^ ^]^ In addition, the flexible self‐assembly of vesicles enables access to the joint display of chimeric receptors, resulting in a broad‐spectrum blocker against multiple CoVs. Prompted by these findings, we engineered hACE2‐ and hDPP4‐overexpressing cell lines as vesicle donors by transfection of hACE2‐iRb3 and hDPP4‐iRb3 carrier plasmid constructs to 293T cells (**Figure** **2**A–C), as previously described methods.^[^ ^15^ ^]^ With the cytomembrane of hACE2‐ and hDPP4‐overexpressing, hACE2‐MV and hDPP4‐MV were fabricated based on the biomimetic vesicle system that we previously developed.^[^ ^9^ ^]^ Next, we generated CoVR‐MVs as antiviral biologics by fusing hACE2‐MVs with hDPP4‐MVs at a ratio of 1:1 in PBS via ultrasonic fusion and membrane extrusion (Figure 2D and Figure S1, Supporting Information)). CoVR‐MVs were negatively charged and showed a typical vesicular structure with a mean diameter of 200 nm (Figure 2E). Dynamic light scattering (DLS) analysis showed that the average size of CoVR‐MVs is 223.67±5.03 nm with PDI at 0.21±0.01 (Figure 2F and Figure S2, Supporting Information). Size measurements established CoVR‐MVs'size stability in PBS for at least 15 d (Figure 2G and Figure S2, Supporting Information). The membrane‐anchored hACE2 and hDPP4 on CoVR‐MVs were detected by Western blotting (Figure 2H). DiD‐labeled hACE2‐MVs and DiO‐labeled hDPP4‐MVs were used to further verify the successful fusion of vesicles. Flow cytometric (FCM) quantification implied an adequate fusion rate of these vesicles as the degree of double‐positive events was as high as 85.8% (Figure 2I and Figure S3, Supporting Information).

To analyze the effects of CoVR‐MVs on viral attachment and binding, we took hACE2 as an example and implemented a competitive inhibition experiment assessing the binding between viral spike protein and CoVR‐MVs. We first quantified the average hACE2 level in CoVR‐MVs utilizing ELISA analysis with soluble hACE2 as a standard. Approximately 5 ng of ACE2 was measured in per µg total protein of CoVR‐MV (Figure S4, Supporting Information). After coincubation with fluorescent protein‐fused SARS‐CoV‐2 spike trimer (STG) and CoVR‐MVs (total protein concentration of 0.2 mg mL^‐1^, hACE2 concentration of 1 µg mL^‐1^) (Figure 2J), the binding of STG and 293T‐hACE2 cells was significantly blocked. On the contrary, MVs without hACE2 display showed no blockade effect, indicating the efficient adsorption of STG by CoVR‐MVs in a hACE2‐dependent manner (Figure 2K). Intriguingly, when co‐incubation with an equivalent concentration of soluble hACE2, the fluorescence signal of STG on 293T‐hACE2 membrane did not decrease significantly (Figure 2K), suggesting the CoVR‐MVs possessed a higher blocking efficiency than soluble hACE2 protein at an equivalent hACE2 concentration. Altogether, the above results demonstrated that CoVR‐MV could serve as a bioinspired scaffold and multimerize receptor molecule with a considerably high affinity of competitive viral binding.

### 2.3 CoVR‐MVs Broadly Interfere with the Infections of SARS‐CoV, MERS‐CoV, SARS‐CoV‐2, and Evolving Variants In Vitro

**Figure 3:**
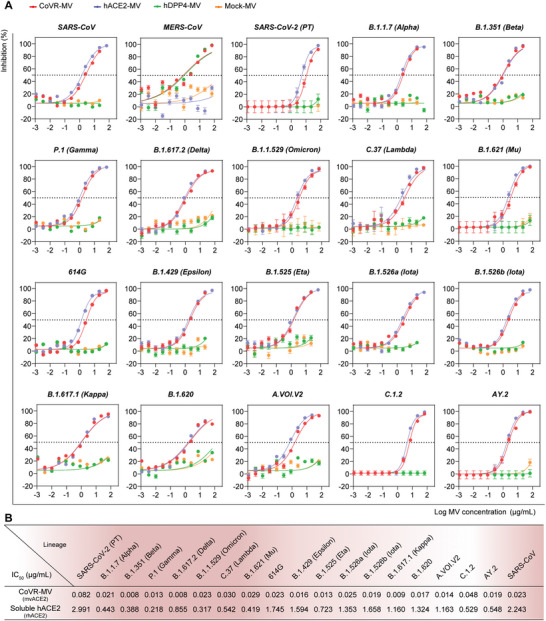
CoVR‐MV broadly interferes with the infections of SARS‐CoV, MERS‐CoV, SARS‐CoV‐2 and evolving variants in vitro. A) Neutralizing curves of CoVR‐MVs and control MVs against SARS‐CoV, MERS‐CoV and 18 different SARS‐CoV‐2 variants in LVpp pseudovirus system (*n* = 3). Y‐axis depicted the percentage of neutralization. Data were shown as mean ± SD. B) List of different viruses and indicated IC_50_ values. IC_50_ values quantified by hACE2 concentration. Soluble hACE2 protein was set as the control.

To determine the virus‐attach efficiency and antiviral spectrum of CoVR‐MVs, we performed neutralization experiments in a previously reported lentivirus‐based pseudovirus particle (LVpp) system that expresses GFP after infection.^[^ ^16^ ^]^ When the LVpp of the three highly pathogenic coronaviruses and 17 SARS‐CoV‐2 variants were utilized, CoVR‐MVs blocked all the test pseudovirus infections in a dose‐dependent manner, as shown by quantitative measurements of GFP expressing cells based on the high‐content screening system in confocal mode (**Figure** **3**A and Figure S5, Supporting Information). In comparison to soluble hACE2, CoVR‐MVs showed 36‐fold higher neutralization capacity against prototype SARS‐CoV‐2 (Figure 3B and Figures S6 and S7, Supporting Information), presumably because of the lipid structure and receptors multivalent display perform some necessary roles in the binding process. Compared to the prototype, CoVR‐MVs remarkably showed 1.7‐ to 10.6‐fold lower IC_50_ against SARS‐CoV‐2 variants, revealing a trend of increasing neutralization titer (Figure 3B and Figure S6, Supporting Information). We compared the antiviral spectrum of CoVR‐MV and an ultrapotent SARS‐CoV‐2 neutralizing antibody S2M11.^[^ ^17^ ^]^ Several variant strains, including B.1.351 (Beta) and B.1.1.529 (Omicron), can fully escape the neutralizing effect of S2M11 (Figure S8, Supporting Information), which further confirms that mutation resistance is a crucial property for antiviral therapy of SARS‐CoV‐2 infection. In addition, CoVR‐MVs did not cause any side effects on the viability of susceptible cells used (Figure S9, Supporting Information). Similar to the results of LVpp experiments, CoVR‐MVs showed high neutralization activity against authentic prototype SARS‐CoV‐2, 614G, B.1.351 (Beta), and B.1.1.529 (Omicron BA.1) variants (Figure S10, Supporting Information). Overall, these data suggested that CoVR‐MVs could broadly interact with the three highly pathogenic coronaviruses and evolving variants, and interfere with their infections.

### 2.4 CoVR‐MVs Therapy Promotes Endogenous Type I IFN Production via Deregulating DHCR7 Inhibition of IRF3 Activation in Macrophages

**Figure 4:**
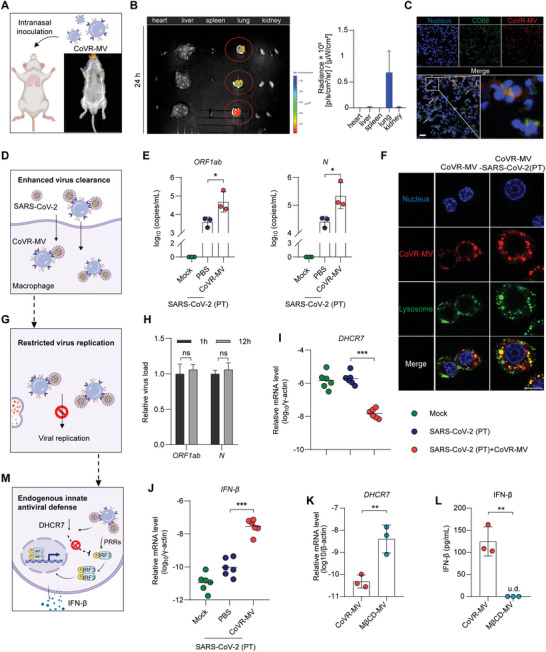
CoVR‐MV therapy promotes endogenous type I IFN production. A) In vivo biodistribution of intranasally administrated DiD‐labeled CoVR‐MVs in the mouse. B) Representative ex vivo fluorescent images and quantitative analysis of major organs from Syrian hamsters at 24 h after intranasal administration with DiD‐labeled CoVR‐MVs (*n* = 3). C) Confocal images showing the capture of CoVR‐MVs by macrophages in lung tissue of mice (Scale bar, 40 µm). D) CoVR‐MV enhance viral clearance via binding SARS‐CoV‐2 and promote the phagocytosis effect of macrophage. E) Viral RNA levels in macrophages collected at 12 h post coincubation (*n* = 3). Viral load was measured by RT‐PCR. The primers of SARS‐CoV‐2 ORF1ab and N genes were used. F) Confocal microscopy images of macrophages stained for DAPI (blue), lysosomes (Lyso‐Tracker, green) incubated with CoVR‐MV (DiD, red) for 3 h (Scale bar, 20 µm). D) After the virus‐CoVR‐MV complex was devoured by macrophage, CoVR‐MV restricts SARS‐CoV‐2 replication. H) Relative viral RNA levels in macrophages collected at 1 h and 12 h post co‐incubation, respectively (*n* = 3). The primers of SARS‐CoV‐2 ORF1ab and N genes were used. Relative mRNA levels of I) *DHCR7* and J) *IFN‐β* in macrophages after co‐incubation of CoVR‐MVs and SARS‐CoV‐2 for 12 h (*n* = 3). The mRNA levels of *DHCR7* and *IFN‐β* were standardized to the house‐keeping gene *γ*‐actin. Relative mRNA levels of K) *DHCR7* in macrophages and L) concentration of IFN‐*β* in supernatant after incubation of CoVR‐MVs or M*β*CD‐MVs for 12 h (*n* = 3). M) CoVR‐MV mediated upregulation of IRF3 and IFN‐*β* by inhibiting DHCR7. Statistical analysis for (E,I,J) were performed using one‐way ANOVA. Statistical analysis for (H,K,L) were performed using unpaired *t*‐test. p‐values <0.05 was considered significant: *P < 0.05, **P <0.01, ***P <0.001, ns indicated no significance to the positive control (*p* > 0.05). u.d. indicated undetectable.

To further investigate the in vivo fate of CoVR‐MVs, Syrian hamsters and mice were intranasally administered with DiD‐labeled CoVR‐MVs (1.5 mg kg^‐1^). We identified that CoVR‐MVs were extensively distributed in the lungs of both hamsters and mice (**Figure** **4**A,B and Figures S11–S13, Supporting Information). The fluorescence signal was undetectable in other organs, including the heart, liver, spleen, kidney, and brain. Furthermore, we found that CoVR‐MVs were present in alveolar macrophages (Figure 4C), which are the primary cells that play an important sentinel role in the lungs by sensing and triggering potent antiviral immunity. Therefore, we set up to investigate the intracellular fate of CoVR‐MVs and SARS‐CoV‐2 in macrophages by an in vitro co‐incubation assay. SARS‐CoV‐2 (1 × 10^6^ TCID_50_) and 200 µg CoVR‐MVs were coincubated with Raw264.7 cells. After 12 h, the increased intracellular viral load suggested that CoVR‐MVs could effectively mediate the internalization of the virus by macrophages (Figure 4D,E). Further confocal imaging revealed the intracellular colocalization of lysosomes and CoVR‐MVs, indicating a possible clearance mechanism of phagocytosis (Figure 4F). In agreement with previous reports,^[^ ^18^ ^]^ we also found that the viral particles delivered by CoVR‐MVs did not cause infection in macrophages (Figure 4G,H).

As the major immune cell type in alveoli, macrophage usually serves as the first line of defense against respiratory virus infections. Recognition of a pathogen by PRRs triggers the secretion of the crucially important type I interferons.^[^ ^19^ ^]^ However, previous reports depict a dysregulated IFN‐I response in SARS‐CoV‐2‐challenged macrophages.^[^ ^20^ ^]^ Intriguingly, we detected the phosphorylation of interferon regulatory factor 3 (IRF3) and production of endogenous IFN‐*β* in macrophages after co‐incubation with CoVR‐MVs and SARS‐CoV‐2 (  Figure S14, Supporting Information). As a biomimetic vesicle derived from a natural cytomembrane, CoVR‐MV is inherently composed of a phospholipid, cholesterol, and membrane proteins. Recently, a critical link between innate immunity and intracellular balance of cholesterol synthesis and import has been unpacked by growing evidence.^[^ ^21^ ^]^ Next, our further research indicated that following the uptake of CoVR‐MVs, the transcription of 7‐dehydrocholesterol reductase (DHCR7) in macrophages was markedly down‐regulated (Figure 4J). As an enzyme that catalyzes 7‐dehydrocholesterol to cholesterol,^[^ ^22^ ^]^ DHCR7 could inhibit IRF3 activation leading to reduce IFN‐I transcription.^[^ ^23^ ^]^ With decreased *DHCR7*, the transcription of *IFN‐β* was significantly up‐regulated in macrophages treated with CoVR‐MVs and SARS‐CoV‐2 (Figure 4I,J). Furthermore, inhibition of DHCR7 by Tamoxifen resulted in a significant increase of *IFN‐β* mRNA level in macrophages with Poly (I:C) stimulation, which confirms that DHCR7 is an important regulator of IFN‐I transcription throughout an infection course (Figure S15, Supporting Information). Intriguingly, solely CoVR‐MVs treatment did not promote transcription of IFN‐*β*, although it reduced the mRNA level of *DHCR7* (Figure S16, Supporting Information), indicating the up‐regulation of *IFN‐β* in macrophages was the joint action of CoVR‐MVs and virus. This is consistent with previous reports that the knockdown of *DHCR7* increases IFN‐I production in virus‐infected cells, but has no effect in virus‐free cells.^[^ ^23^ ^]^ Furthermore, treatment of vesicles (M*β*CD‐MV) that derived from the cholesterol‐removing cell membrane failed to significantly decrease the mRNA level of *DHCR7* in macrophages (Figure 4K). Meanwhile, the IFN‐*β* level was not significantly elevated (Figure 4L). These results suggested that the cholesterol of CoVR‐MVs was a potentially important factor to enhance IFN‐*β* production by suppressing DHCR7 (Figure 4M). Taken together, these results delineated the process of CoVR‐MVs‐mediated viral clearance and promotion of endogenous IFN‐I in macrophages, and provided some evidence that cholesterol of CoVR‐MVs might play a potentially critical role.

### 2.5 CoVR‐MVs Reverse the Imbalanced Innate Immune Responses in SARS‐CoV‐2 Infected Syrian Hamsters

**Figure 5:**
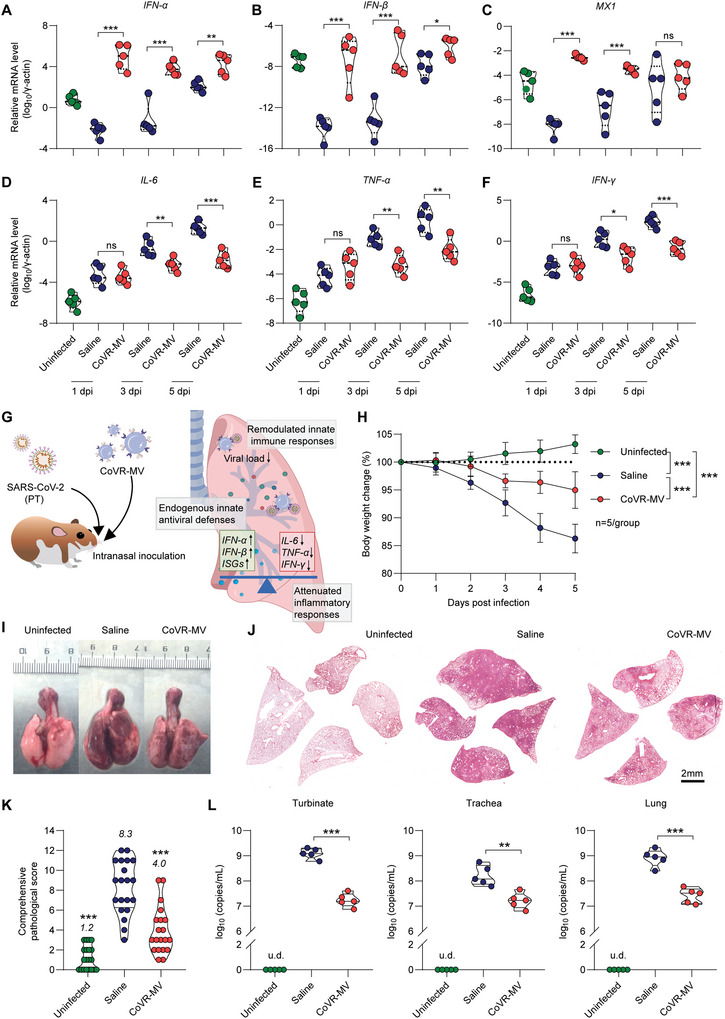
CoVR‐MV reverses the imbalanced innate immune responses in SARS‐CoV‐2 infected Syrian hamsters. Relative mRNA levels of A) *IFN‐α*, B) *IFN‐β* and C) *MX1*, and proinflammatory cytokines include D) IL‐6, E) TNF‐*α* and (F) IFN‐*γ* in the lung tissues collected from euthanized hamsters at 1, 3, and 5 dpi, respectively (*n* = 5). The mRNA levels were standardized to the house‐keeping gene *γ*‐actin. G) Schematic illustration for CoVR‐MVs mediated rebalance of the dysregulated innate immune response. H) Body weight changes of the survived hamsters from 0 to 5 dpi were recorded (*n* = 5). Representative I) gross images and J) H&E staining images for lung lobes collected at 5 dpi were shown. Scale bar = 2 mm. H&E staining images for all of the remaining hamster lobes were shown in Figure S13 (Supporting Information). K) Comprehensive pathological scores for lung sections were determined based on the severity and percentage of injured areas for each lung lobe (details shown in Table S1, Supporting Information). L) Viral RNA levels in turbinate, trachea and lung tissues collected at 5 dpi were measured by RT‐PCR (*n* = 5). The primers of SARS‐CoV‐2 ORF1ab gene were used. Data were shown as mean ± SD. Statistical analysis for H) was performed using two‐way ANOVA. All other analyses were conducted using one‐way ANOVA. *p*‐values <0.05 was considered significant: *P < 0.05, **P < 0.01, ***P < 0.001, ns indicated no significance to the positive control (*p* > 0.05). u.d. indicated undetectable.

A dose‐gradient regimen demonstrated that a high dose (1.5 mg kg^‐1^) of inhaled CoVR‐MV therapy at 0 dpi is adequate to alleviate the severity of COVID‐19 at 5 dpi, rather than the lower doses of 0.3 and 0.06 mg kg^‐1^ (Figure S17, Supporting Information). In addition, followed‐up inhaled CoVR‐MV therapies from 1 to 4 dpi can improve the therapeutic effect (Figure S17, Supporting Information). Next, we investigated the immunomodulatory effects of CoVR‐MVs in vivo. Syrian hamsters were intranasally inoculated with 1 × 10^4^ PFU of prototype SARS‐CoV‐2. Afterward, hamsters received inhaled CoVR‐MVs (1.5 mg kg^‐1^) therapy at 0, 1, and 3 dpi, respectively. Uninfected hamsters and SARS‐CoV‐2‐infected ones with inhaled saline therapy were set as control groups. Lung tissues were collected from euthanized hamsters at 1, 3, and 5 dpi, respectively, for analysis of innate immune response. The mRNA levels of typical pro‐inflammatory cytokines, type I IFN and ISG in homogenized lung tissues were measured by RT‐PCR. Similar to the results of Figure 1, SARS‐CoV‐2 induced an imbalanced innate immune response that characterized by transcriptional suppression of antiviral genes (**Figure** **5**A–C and Figure S18, Supporting Information), and high mRNA levels of *IL‐6*, *IFN‐γ*, *TNF‐α* and *NFκB p65/p65* (Figure 5D–F and Figure S19, Supporting Information). The ELISA results of *IL‐6* and *IFN‐γ* concentrations in lung homogenate samples further confirm the excessive release of proinflammatory cytokines (Figure S20, Supporting Information). This defectiveness of the antiviral immune response is particularly significant in the early stage of infection. Importantly, in contrast to the saline‐treated hamsters, a single dose of inhaled CoVR‐MVs therapy caused approximately 1000 000‐, 100 000‐ and 50000‐fold increases of *IFN‐α*, *IFN‐β*, *MX1*, *ISG15*, *ISG20*, *ISG56* and *OAS1* mRNA levels at 1 dpi, respectively (Figure 5A–C and Figure S18, Supporting Information), which were even higher than those of uninfected healthy controls. Of note, this finding indicated an early activation of endogenous antiviral responses. Additionally, subsequent inhaled CoVR‐MVs therapy at 1 and 3 dpi maintained the trends of type I IFN signaling activation and pro‐inflammatory suppression to 5 dpi. Based on these results, we speculated that the re‐modulation of imbalanced immune response by a single dose of CoVR‐MVs might effectively ameliorate lung pathology and control virus load (Figure 5G). Indeed, the results of the virological and pathological analysis confirmed that a single dose of inhaled CoVR‐MVs therapy (1.5 mg kg^‐1^) when inoculation of 1 × 10^4^ PFU of prototype SARS‐CoV‐2 can vastly decrease body weight loss (Figure 5H) and suppressed diffuse lung injury in lung tissues in hamsters (Figure 5I–K, Figure S21 and Table S1, Supporting Information). Notably, CoVR‐MVs therapy showed approximately 10‐ to 50‐fold decrease of viral RNA in turbinate, trachea, and lung, respectively (Figure 5L). Collectively, the results suggested that CoVR‐MVs could re‐modulate host innate immune responses to SARS‐CoV‐2 in vivo, and effectively protect hamsters from cytokine storm and subsequent lung pathogenic changes.

### 2.6 Inhaled CoVR‐MVs Therapy Rescues Lethal ARDS in Syrian Hamsters Infected with SARS‐CoV‐2 Variants

**Figure 6:**
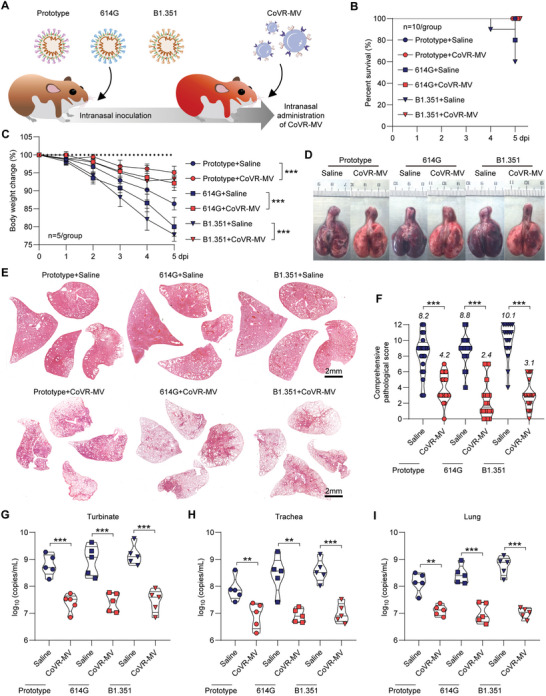
CoVR‐MV protects hamsters from lethal ARDS of SARS‐CoV‐2 variant infections. A) Schematic diagram of SARS‐CoV‐2 infection and CoVR‐MVs therapy. Hamsters were intranasally inoculated with 1 × 10^4^ PFU of prototype SARS‐CoV‐2, 614G, and B.1.351 variants, respectively. The infected hamsters treated with saline were set as controls. B) Survival analysis (*n* = 10). C) Body weight changes of the survived hamsters from 0 to 5 dpi were recorded (*n* = 5). Representative D) gross images and E) H&E staining images for lung lobes collected at 5 dpi were shown. Scale bar = 2 mm. H&E staining images for all of the remaining hamster lobes were shown in Figure S15 (Supporting Information). F) Comprehensive pathological scores for lung sections were determined based on the severity and percentage of injured areas for each lung lobe (details shown in Table S2, Supporting Information). Viral RNA levels in G) turbinate, H) trachea and I) lung tissues collected at 5 dpi were measured by RT‐PCR (*n* = 5). The primers of SARS‐CoV‐2 ORF1ab gene were used. Data were shown as mean ± SD. Statistical analysis for C) was performed using two‐way ANOVA. All other analyses were conducted using one‐way ANOVA. *p*‐values <0.05 was considered significant: *P < 0.05, **P < 0.01, ***P < 0.001, ns indicated no significance to the positive control (*p*> 0.05). u.d. indicated undetectable.

SARS‐CoV‐2 variants with higher transmissibility and pathogenicity pose a great challenge to the effectiveness of therapy. The 614G and B.1.351 strains were demonstrated to acquire higher infectivity and lethality than the prototype SARS‐CoV‐2.^[^ ^24^ ^]^ Thus, we asked whether CoVR‐MVs therapy can efficiently rescue the hamsters after SARS‐CoV‐2 variant infection at a lethal dose. To that end, hamsters were respectively infected with 1×10^4^ plaque‐forming units (PFU) of prototype SARS‐CoV‐2, 614G, and B.1.351 strains, followed by treatment with intranasal CoVR‐MVs once per day for 5 d (**Figure** **6**A). H&E staining results of the main organs demonstrated the safety of inhaled CoVR‐MVs (Figure S22, Supporting Information). Survival rate and body weight loss were recorded from 0 to 5 dpi. As a result, two out of ten hamsters died after the 614G infection and four out of ten hamsters died after the B.1.351 infection within 5 d (Figure 6B). In contrast, all of the hamsters with CoVR‐MVs therapy survived at 5 dpi (Figure 6B). Then, all of the survived hamsters were euthanized for immunological and virological examinations. Inhaled CoVR‐MVs therapy was adequate to alleviate the typical disease manifestations of COVID‐19, such as body weight loss (Figure 6C), diffused lung injury (Figure 6D–F, Figure S23 and Table S2, Supporting Information) and viral replication (Figure 6G–I) in the hamsters infected with three different SARS‐CoV‐2 variant strains. Collectively, these results confirmed that CoVR‐MVs ameliorate the disease outcome of COVID‐19 in hamsters after a lethal dose infection of SARS‐CoV‐2 variants. More importantly, the therapeutic efficiency of CoVR‐MVs would not be attenuated by the elevated pathogenicity in different SARS‐CoV‐2 variants.

## 3 Discussion

The three highly pathogenic coronaviruses (SARS‐CoV, MERS‐CoV, and SARS‐CoV‐2) that have caused pandemics in this century have emerged as one of the most significant public health challenges and impose a great threat to human health. As with SARS‐CoV and MERS‐CoV, the important understanding of the delicate balance of the viral‐host interaction that is responsible for COVID‐19 is now increasingly appreciated: 1) fast and robust initial viral replication in respiratory tract organs; 2) early inhibition of IFN production and signaling causes a defective antiviral innate immune response which delays viral clearance and drives immunopathology; 3) imbalanced innate immune response usually induces the excessive release of proinflammatory cytokines, which indicates a lousy disease outcome; and lastly 4) cytokine storm and diffuse lung tissue injury largely increase the risk of lethal ARDS.^[^ ^25^ ^]^ Therefore, achieving a rebalanced interplay between virus and host innate immunity is a crucial route to developing therapeutic strategies against highly pathogenic coronavirus‐caused ARDS.

In the past few years, we devised receptor‐based nanodecoys to combat hepatitis B virus infection.^[^ ^9^ ^]^ To date, the receptor‐mediated antiviral effect of nanomedicines has been widely demonstrated in the studies of HIV,^[^ ^26^ ^]^ ZIKV,^[^ ^27^ ^]^ and SARS‐CoV‐2.^[^ ^28^ ^]^ As a kind of novel biomaterial, cytomembrane‐based biomimetic vesicles continue to receive considerable attention in antiviral therapy owing to the remarkable ability to interact with biological molecules, while their in vivo behavior and interplay with the immune system have been often ignored but are now increasingly appreciated. In this study, we have developed an antiviral platform (CoVR‐MVs) and revealed the multidimensional mechanisms of CoVR‐MVs in vivo. By taking advantage of the maximized spike protein target interface, the CoVR‐MVs platform shows notable promise to broadly absorb SARS‐CoV, MERS‐CoV, SARS‐CoV‐2 and its circulating variants (Figures 2 and 3). As coronaviruses usually mutate over time and escape from existing neutralizing antibodies, the CoVR‐MVs platform provides a new option for broad‐spectrum antiviral agents. In comparison to soluble hACE2, CoVR‐MV showed a higher neutralization capacity against SARS‐CoV‐2 (Figure 3B and Figure S8, Supporting Information), presumably because the natural vesicular structure and composition of CoVR‐MV perform some necessary roles in the binding process. The lipid environment is well‐suited to control the membrane‐bound receptor's conformation and aggregation, which maximizes their binding affinity and amount of virus‐decoy binding.^[^ ^29^ ^]^ Another potential advantage of CoVR‐MV is the necessary host attachment factors, such as heparan sulfate^[^ ^30^ ^]^ and TMPRSS2,^[^ ^31^ ^]^ which are naturally expressed on the host cell membranes and can largely promote the virus‐receptor binding efficiency. This unique natural structure and feature enable CoVR‐MV to overcome the disadvantages of small molecular drugs, such as lack of targeting and direct neutralization effect.

Next, we analyzed that the effector immune cells that are responsible for clearing CoVR‐MVs are alveolar macrophages. Due to the inherent interaction with phagocytes, the designed nanoscale CoVR‐MVs administered intranasally could also break the general mode of action of direct‐acting antivirals, increase the efficiency of macrophage‐mediated viral clearance, and reverse the imbalanced innate immune responses (Figures 4 and 5). Conventional antivirals and anti‐inflammatory drugs often fail to trigger a sufficient endogenous antiviral immune response due to multiple mechanisms that coronavirus developed to block IFN‐I production and signaling. In this study, we further found the ability of the CoVR‐MV‐virus complex in promoting IFN‐I production and signaling. Moreover, the cholesterol of CoVR‐MVs is essential in this process. The intracellular balance of cholesterol synthesis and import plays an essential role in regulating immune cell activation. Generally, cholesterol is delivered into cells by low‐density lipoprotein and is gradually into free cholesterol through the endo‐lysosomal system. Based on the colocalization of lysosomes and CoVR‐MVs in macrophages, we speculated that cholesterol delivered by CoVR‐MVs affected the synthesis of intracellular cholesterol, which was reflected in the down‐regulation of *DHCR7*. DHCR7 is responsible for catalyzing 7‐dehydrocholesterol to cholesterol in the final step in the biogenesis of cholesterol. It has been demonstrated that silencing *DHCR7* by RNAi in macrophages can increase the activation of IRF3. Although we provide some preliminary evidence for the potential role of cholesterol delivered by CoVR‐MVs in immunomodulation, the detailed mechanism needs further investigation.

In summary, our results provide insight into how biomimetic antiviral biologics can significantly reverse imbalanced innate immune responses and rescue Syrian hamsters from highly pathogenic coronavirus‐caused lethal pneumonia by interacting with the virus (Figure 6). In comparison with previous studies, this investigation revealed the multidimensional effects of CoVR‐MVs in vivo, including the intervention on viral infection and reconstruction of a spontaneous antiviral immune response, which further improves our understanding of the antiviral mechanisms of allied biologics. We envision that the described strategy of turning the imbalanced innate immunity from inflammatory to antiviral in a highly bionic and self‐sustainable manner can be widely deployed to defend other virus families with high mutation rates and fairly strong immunepathogenicity.

## 4 Experimental Section

### Cell Culture

The cell lines of 293T, H1299, H1299‐hACE2, 293T‐hACE2, and 293T‐hDPP4 were cultured in Dulbecco's modified Eagle medium (DMEM) (#D6429, Sigma) supplemented with 10% fetal bovine serum (#10099‐141, Thermo Scientific), 0.1 × 10^−3^ m nonessential amino acids (#1140‐050, Thermo Scientific), at 37 °C and 5% CO_2_ in a humidified incubator. To ensure the stable expression of transfected constructs in cells, the culture medium was supplemented with blasticidin (10 µg mL^‐1^) for H1299‐hACE2, and puromycin (1 µg mL^‐1^) for 293T‐hACE2, respectively.

### Generation of CoVR‐MVs

The cell membrane was collected by a repeated freeze‐thaw process as previously reported.^[^ ^9^ ^]^ Cell suspensions in phosphate buffered saline (PBS) were frozen at ‐80 °C, thawed at room temperature, and pelleted by centrifugation at 1000 *g* for 5 min. Following three repeated washes with cold PBS mixed with protease inhibitor cocktail (#87785, Thermo Scientific), cell membranes were suspended in cold PBS and sonicated in a sterile 1.5 mL EP tube under lower power (22.5 W, 20 s ×3) on ice. Then the vesicles were isolated by multi‐steps of differential centrifugation before being resuspended in PBS. The product was then introduced to a Mini‐Extruder (Avanti Polar Lipids) equilibrated in PBS (200 nm pore‐sized membrane filters) to further purify uniform nanovesicles. Vesicles were stored at 4 °C for 1 week or placed in long‐term storage at ‐80 °C. Total membrane protein content was quantified by a BCA protein assay kit (#23227, Thermo Scientific). For CoVR‐MV generation, hACE2‐MVs and hDPP4‐MVs were mixed and then extruded through 200‐nm pores on the Mini‐Extruder (Avanti Polar Lipids).

### Characterization of CoVR‐MVs

The size and surface zeta potential of CoVR‐MV and other vesicles were measured by dynamic light scattering (DLS) using a Malvern Zetasizer Nano‐ZS90. For electron microscopy visualization, CoVR‐MVs samples were negatively stained with freshly filtered 2% uranyl acetate. Grids were examined with a transmission electron microscope (FEI Tecnai G2 Spirit) at an accelerating voltage of 120 kV and photographed at a magnification of 25000. The membrane‐anchored hACE2 and hDPP4 on CoVR‐MV was detected by western blot. The content of hACE2 of CoVR‐MVs was analyzed with hACE2 (#ab235649, Abcam) ELISA kits according to the manufacturer's instructions.

### Cell Imaging Assay

For direct visualization of the cellular binding of spike proteins, the 293T‐hACE2 cells were seeded at 2 × 10^4^ cells per well in poly‐d‐lysine pretreated CellCarrier‐96 Black plate. After 1 d culture, the fluorescent probes were added to the cell cultures. Ensure a final concentration of 2.5 × 10^−9^ m for S‐ectodomain trimer (ST)‐based protein probes (designated as STG) in culture medium. Cell images with multichannel fluorescence (STG, Ex:488/Em:525; hACE2‐mRuby3, Ex:561/Em:590; H2BiRFP670, Ex:640/Em670) were acquired (Opera Phenix) at 1 h after probe loading in wash‐free and live‐cell conditions.

### Neutralization Assay of Pseudotyped Virus System

The lentiviral pseudotyping particles (LVpp) of SARS‐CoV, MERS‐CoV, SARS‐CoV‐2 and its variant strains were produced as previously described.^[^ ^16^ ^]^ For determinations of CoVR‐MV mediated inhibition for LVpp infection, the plated H1299‐hACE2 cells were incubated with LVpp inoculum (0.5 TU per cell) and culture media containing the indicated concentration of Mock‐MV, hACE2‐MV, hDPP4‐MV or CoVR‐MV at 37 °C for 48 h. Then the fluorescent imaging analysis and IC50 calculations were based on the infection inhibition ratio of serial dilutions and determined by the 4‐parameter logistic (4PL) regression using GraphPad Prism 8.0.

### Experimental Animal and Biosafety

Six‐ to eight week male hamster golden Syrian hamsters were raised in specific pathogen‐free animal feeding facilities. All the animal experiments were approved by the Medical Ethics Committee of Joint Institute of Virology of Shantou University and The University of Hong Kong (SUCM2021‐112). All experiments with infectious SARS‐CoV‐2 were performed in the biosafety level 3 (BSL‐3) and animal biosafety level 3 (ABSL‐3) facilities affiliated with the State Key Laboratory of Emerging Infectious Diseases, School of Public Health, The University of Hong Kong. The staffs wear powered air‐purifying respirators that filter the air, and disposable coveralls when they culture the virus and handle animals that are in isolators. The researchers in this personal protective equipment are disinfected before they leave the room and then shower on exiting the facility. All facilities, procedures, training records, safety drills, and inventory records are subject to periodic inspections and ongoing oversight by the institutional biosafety officers who consult frequently with the facility managers.

### Preparation of Virus Stock

The SARS‐CoV‐2 prototype strain AP‐8 (hCoV‐19/China/AP8/2020; GISAID accession number: EPI_ISL_1655937), the 614G mutation virus strain AP‐62 (hCoV19/China/AP62/2020; GISAID accession number: EPI_ISL_2779638), the B.1.351 (Beta) variant strain AP‐100 (hCoV19/China/AP100/2021; GISAID accession number: EPI_ISL_2779639) and the B.1.1.529 (Omicron) variant strain AP‐309 (shares a total similar sequence with hCoV19/USA/FL‐CDC‐STM‐V8T65K8SS/2021; GISAID accession number: EPI_ISL_8182026) were passaged on Vero cells (#CCL‐81, ATCC). Viral stocks were prepared in Vero cells with DMEM containing 2% FBS, 5 µg mL^‐1^ TPCK‐trypsin, penicillin–streptomycin and 30 mmol L^‐1^ MgCl_2_ (#11995, #10270106, #T1426 and #15140‐122; purchased from GIBCO, Sigma‐Aldrich and Invitrogen). Approximately 200 mL of supernatant was collected from several culture flasks and centrifuged to remove cell fragments. After that, the supernatant was further purified and concentrated and by a centrifugal filter device. Finally, approximately 50 mL of viral stock was obtained. The titer of live virus particle was detected by a standard plaque assay in Vero cells. The virus stocks were stored in ultra‐low temperature refrigerators.

### Neutralization Assay of Authentic Virus System

Neutralization assay of authentic SARS‐CoV‐2 viruses was measured by a standard median tissue culture infective dose (TCID_50_) method in Vero cells seeded in a 96‐well plate as previously described.^[^ ^12^ ^]^ Samples were serially diluted and incubated with 100 TCID_50_ of SARS‐CoV‐2 for 1 h. And then, the mix was added into a 96‐well plate seeded with Vero cells for another 1 h incubation. After that, 3 d after incubation, the inhibition of cytopathic effects was observed and used for the calculation of IC_50_.

### Virus Inoculation and Sample Collection in Hamster Model

The hamsters were anesthetized by isoflurane (#R510‐22, RWD Life Science) and then nasally inoculated with 1 × 10^4^ PFU dose of SARS‐CoV‐2 diluted in 200 µL PBS (#10010031, GIBCO). The ARDS animal model that caused by SARS‐CoV‐2 infection has disease features include body weight loss, diffuse lung injury, high viral load in respiratory tract organs include turbinate, trachea, and lung. A part of the critical cases might die within 5 d. The body weight of these hamsters was measured by electronic balance. Hamsters were treated with isoflurane lightly. After that, capillary tube was used to collect blood from orbital vein. Hamsters were euthanized at indicated time point for detection of viral load and analysis of pathogenesis in lung lobes.

### Detection of Viral RNA

Viral RNA was extracted by using a QIAamp Viral RNA Mini kit (#52906, Qiagen) according to the manufacturer's instructions. The RT‐PCR was conducted by using the SLAN‐96S Real‐Time System (Hongshi, Shanghai, China) with a SARS‐CoV‐2 RT‐PCR Kit from Wantai (Beijing, China). Relative Viral RNA of SARS‐CoV‐2 ORF1ab gene and NP gene were determined using primer pairs and probes shown in the kit instruction. Viral RNA copies were expressed on a log_10_ scale after normalized to the standard curve obtained by using ten‐fold dilutions of a SARS‐CoV‐2 stock.

### Detection of Cytokine in mRNA and Protein Levels

The lung tissues were cleaved into small pieces and soaked in RNAlater (#AM7021, Invitrogen). Total RNAs in lysed lung tissues were extracted with RNeasy Mini kit (#74106, Qiagen) and reverse‐transcribed to cDNA with Fast‐King Strand cDNA Synthesis Kit (#FP313, TIANGEN, Beijing). Diluted cDNAs (1:10) were quantified using SYBR Green I‐based real‐time PCR using the LightCycler 480 instrument (Roche) per the manufacturer's instructions. The threshold cycle (Ct) of each gene was normalized to the internal reference gene (hamster *γ*‐actin) and the comparative Ct (2‐ΔΔCt) method was utilized to calculate changes in chemokine and cytokine gene expression profile. ELISA Kits were used to detect the concentration of IL‐6 (#CSB‐E14304HA, Cusabio) and IFN‐*γ* (#CSB‐EL011050HA, Cusabio) in lung tissue homogenate.

### Histopathological Studies

For pathological analysis, lung tissues were fixed in formalin for more than 48 h, dehydrated and then embedded in paraffin wax. The wax block of lung tissues was cut into 4 µm sections for several pathological staining and analysis. H&E staining was employed for analysis of general lung pathogenic lesions, including pulmonary edema, consolidation and inflammation. The standards for pathological score of lung tissues in this study are derived from the previous study in a hamster model.^[^ ^12^ ^]^ Comprehensive pathological score of lung sections was performed according to the degree of lung lesions, including alveolar septum hyperplasia, consolidation and impairment of alveolar structure, fluid exudation, mucus suppository, thrombus, inflammation recruitment and infiltration of immune cells in each individual lung lobe. For each hamster, three or four lung lobes were employed for evaluation of comprehensive pathological score. In brief, the H&E staining result of each lung lobe was analyzed for its severity of pathological change. The pathological score included: a) Alveolar septum thickening and consolidation; b) Hemorrhage, exudation, pulmonary edema, and mucous; c) Recruitment and infiltration of inflammatory immune cells. For each issue, scores related to the severity were as follows: 0 indicated no pathological change was observed, 1 indicated moderate pathological change, 2 indicated mild pathological change, 3 indicated severe pathological change and 4 indicated very severe pathological change. In conclusion, scores of such three issues were added as the comprehensive pathological score of a lung lobe, and the average comprehensive pathological score of the lobes indicated the severity of lung pathogenesis in an evaluated hamster. The images of whole lung lobes were screened by a high‐throughput screening microscope system (EVOS M7000, Invitrogen of Thermo Fisher Scientific).

### Statistical Analysis

The statistical analysis relied on one‐way ANOVA, two‐way ANOVA, and unpaired two‐tailed *t*‐test methods. Data are presented as the median or means ± SD. p‐values <0.05 were considered significant: *P <0.05, **P < 0.01, ***P <0.001, ns indicates no significance.

## Conflict of Interest

The authors declare no conflict of interest.

## Supporting information

Supporting Information

## Data Availability

The data that support the findings of this study are available in the supplementary material of this article.
