# Supplementary material for: Antiviral Nanobiologic Therapy Remodulates Innate Immune Responses to Highly Pathogenic Coronavirus
Source: Adv Sci (Weinh). 2023 Apr 25;10(17):2207249. doi: 10.1002/advs.202207249 (PMC10265047; doi:10.1002/advs.202207249)
Supplement: Supplementary file 1 — Supporting Information [file ADVS-10-2207249-s001.pdf]

ADVANCED SCIENCE
Open Access

Supporting Information

for *Adv. Sci.*, DOI 10.1002/advs.202207249

Antiviral Nanobiologic Therapy Remodulates Innate Immune Responses to Highly Pathogenic Coronavirus

Xuan Liu, Lunzhi Yuan, Jijing Chen, Yali Zhang, Peiwen Chen, Ming Zhou, Jiaxuan Xie, Jian Ma, Jianzhong Zhang, Kun Wu, Qiyi Tang, Quan Yuan, Huachen Zhu, Tong Cheng*, Yi Guan*, Gang Liu* and Ningshao Xia**

Supporting Information

Antiviral nanobiologic therapy re-modulates innate immune responses to highly pathogenic coronavirus

Xuan Liu, Lunzhi Yuan, Jijing Chen, Yali Zhang, Peiwen Chen, Ming Zhou, Jiaxuan Xie, Jian Ma, Jianzhong Zhang, Kun Wu, Qiyi Tang, Quan Yuan, Huachen Zhu, Tong Cheng*, Yi Guan*, Gang Liu*, Ningshao Xia**

*Corresponding author. Email: zhuhch@hku.hk (H.Z.), tcheng@xmu.edu.cn (T. C.), yguan@hku.hk (Y.G.), gangliu.cmitm@xmu.edu.cn (G.L.), nsxia@xmu.edu.cn (N.X.)

This PDF file includes:

Supplementary Figures 1 to 23
Supplementary Tables 1 to 3

Supplementary Figures

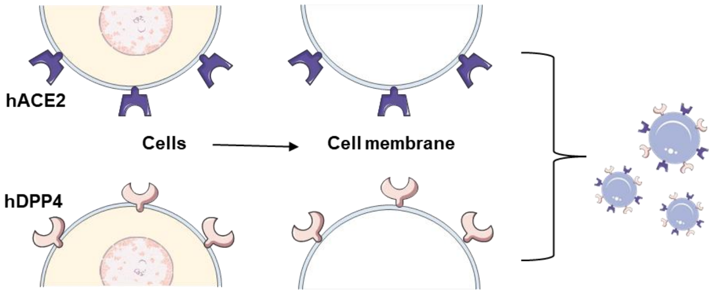

Figure S1. Schematic illustration for CoVR-MV displaying abundant hACE2 and hDPP4 receptors.

A

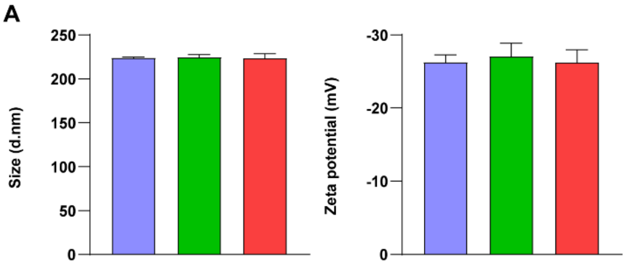

	hACE2-MV	hDPP4-MV	CoVR-MV
Size (nm)	223.83±1.02	224.83±2.89	223.67±5.03
PDI	0.21±0.04	0.22±0.02	0.21±0.01
Zate (mV)	-26.27±1.03	-27.07±1.80	-26.23±1.74

B

Days	3	6	9	12	15
Size (nm)	225.07 ±5.16	221.90±2.52	226.70±2.74	221.13±4.12	230.00±2.52
PDI	0.20±0.04	0.22±0.02	0.23±0.01	0.22±0.01	0.22±0.03

Figure S2. Preparation and characterization of CoVR-MV. A) Mean size, PDI value and zeta potential of hACE2-MV, hDPP4-MV and CoVR-MV. B) Mean size and PDI value of CoVR-MV after 3, 6, 9, 12 and 15 days of storage at 4°C. Data were shown as mean ± SD, n=3 independent experiments.

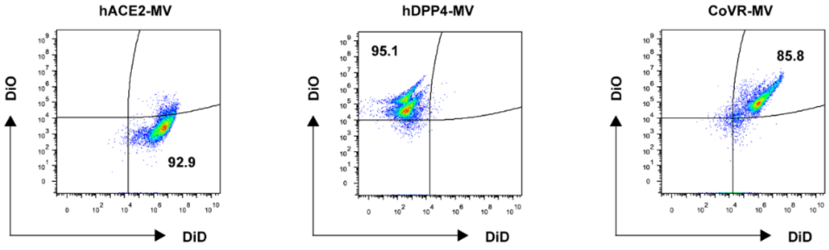

Figure S3. Flow cytometry-based assay demonstrated a successful assembly of chimeric-receptors vesicles. hACE2-MV and hDPP4-MV were labeled with DiD and DiO dyes, respectively.

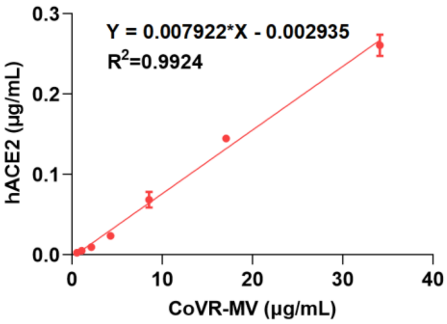

Figure S4. hACE2 protein level of CoVR-MV analyzed by ELISA.

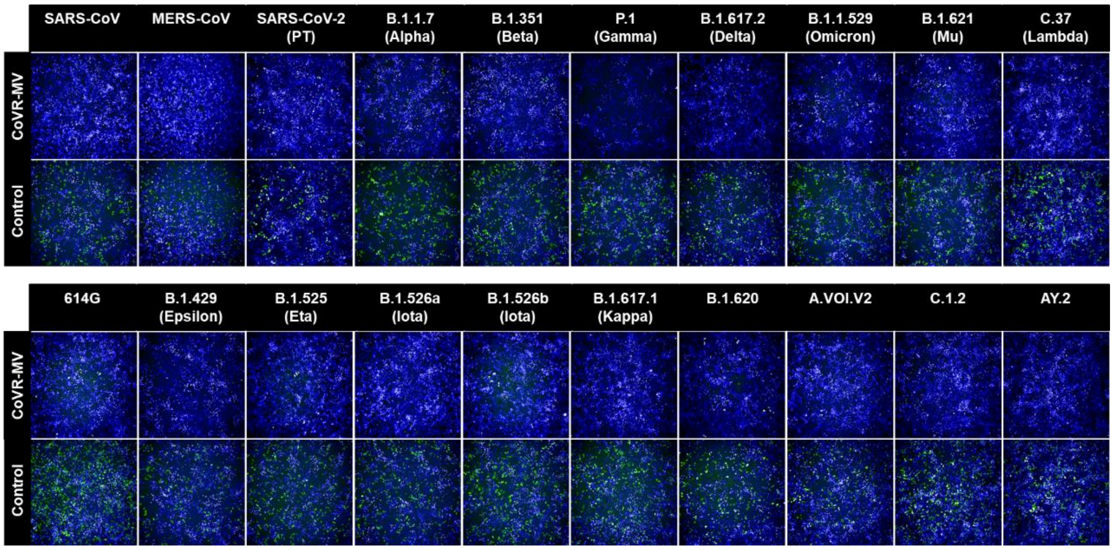

Figure S5. CoVR-MV significantly blocked the infection of SARS-CoV, MERS-CoV, SARS-CoV-2 and the variants in the pseudovirus system.

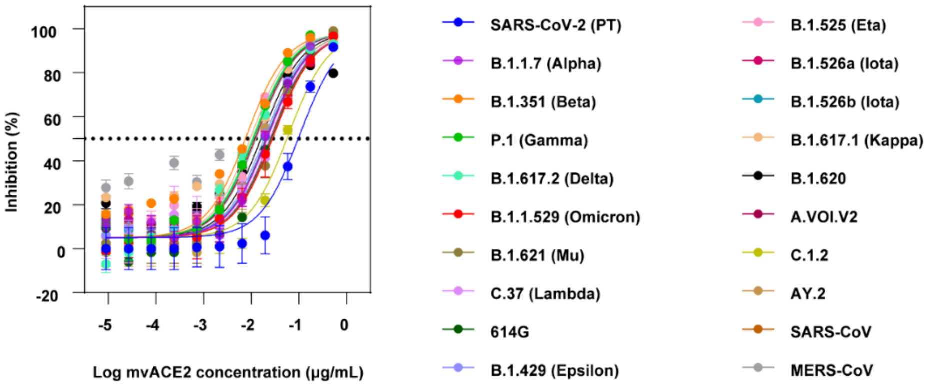

Figure S6. CoVR-MV blocked the infection of SARS-CoV, MERS-CoV, SARS-CoV-2 and the variants in the pseudovirus system. Data were shown as mean ± SD, n=3 independent experiments.

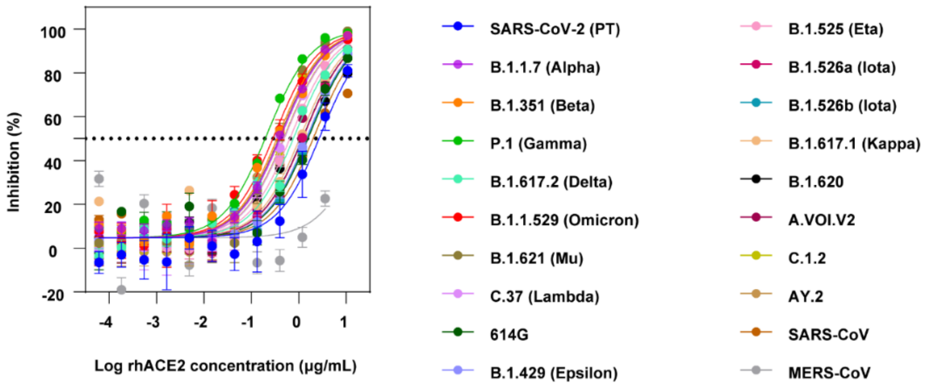

Figure S7. Soluble hACE2 blocked the infection of SARS-CoV, SARS-CoV-2 and the variants in the pseudovirus system, but was unable to block MERS-CoV. Data were shown as mean ± SD, n=3 independent experiments.

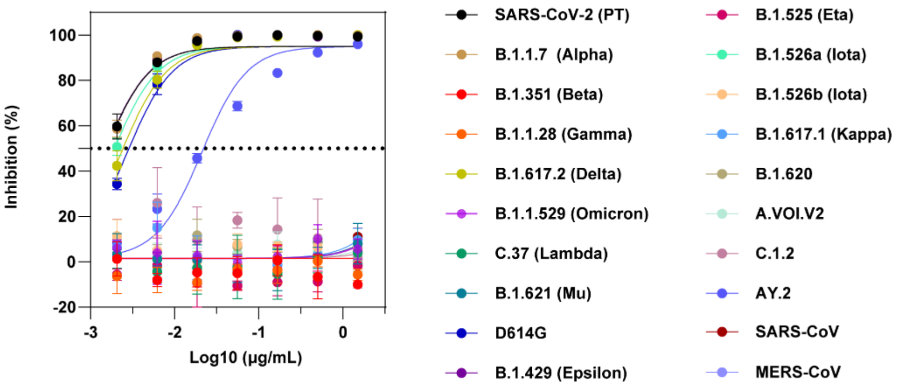

Figure S8. Neutralization of SARS-CoV-2 by antibody S2M11. Data was shown as mean ± SD, n=3 independent experiments.

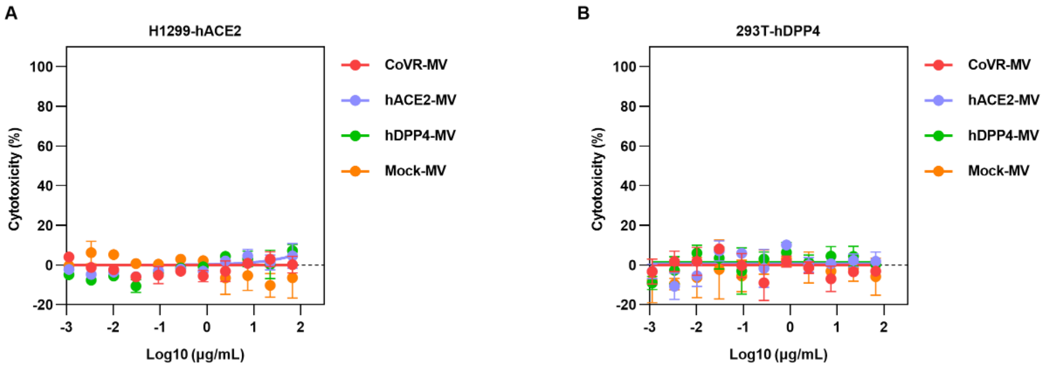

Figure S9. Cytotoxicity of CoVR-MV, hACE2-MV, hDPP4-MV and Mock-MV in (A) H1299-hACE2 and (B) 293T-hDPP4 cells. Data were shown as mean ± SD, n=3 independent experiments.

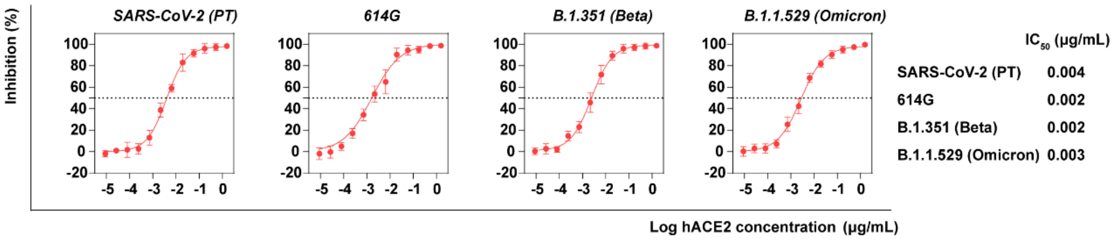

Figure S10. Validation for the broad-spectrum neutralization of CoVR-MV against authentic SARS-CoV-2 and the variant strains *in vitro*. CoVR-MV was serially diluted, and incubated with authentic SARS-CoV-2 prototype (PT) strain, 614G strain and B.1.351 strain. Neutralizing curves of CoVR-MV against SARS-CoV-2 PT strain, 614G strain, B.1.351 (Beta) strain and B.1.1.529 (Omicron) strain. The inhibition efficiency was measured by IC_{50} values, respectively. Data were shown as mean ± SD, n=3 independent experiments.

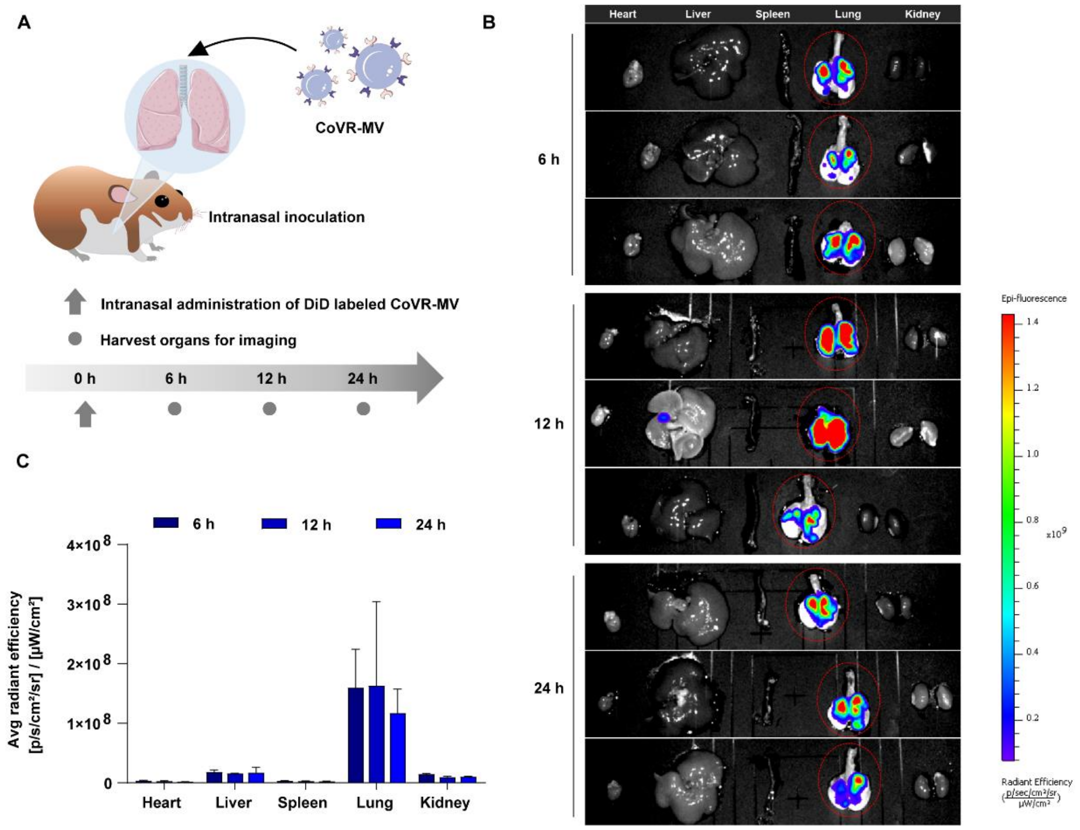

Figure S11. Biodistribution of intranasally administrated CoVR-MV in the Syrian hamster. A) Schematic diagram of the experimental design. B) *Ex vivo* fluorescent images and (C) quantitative analysis of major organs from Syrian hamsters at 6, 12, and 24 hours after intranasal administration with DiD-labelled CoVR-MV. The graph showed the accumulation of DiD-labelled CoVR-MV in the lungs after intranasal administration. Data were shown as mean ± SD, n= 3 independent experiments.

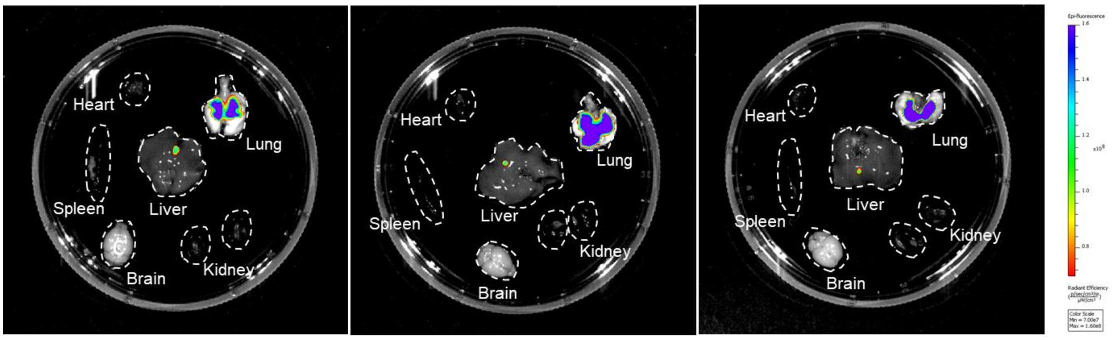

Figure S12. Representative *Ex vivo* fluorescent images of major organs from mice at 12 hours after intranasal administration with DiD-labelled CoVR-MV.

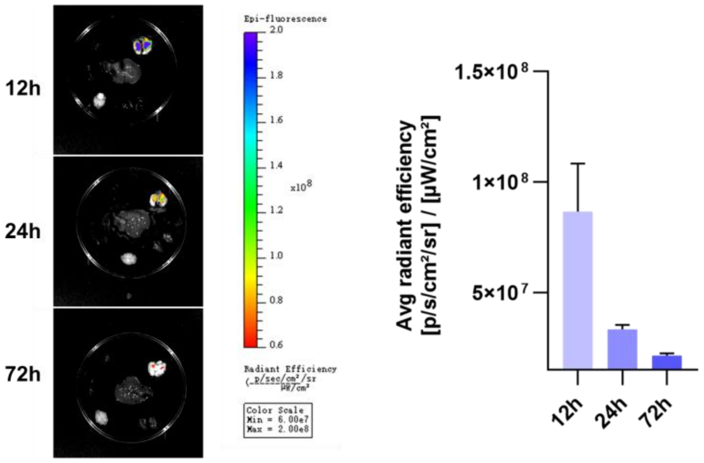

Figure S13. Representative *Ex vivo* fluorescent images and quantitative analysis of major organs from mice at 12, 24, and 72 hours after intranasal administration with DiD-labelled CoVR-MV (1.5 mg/kg). Data were shown as mean ± SD, n= 3 independent experiments.

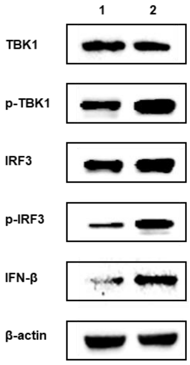

Figure S14. Western blot analysis of expressions of TBK1, p-TBK1, IRF3, p-IRF3 and IFN-β in macrophages at 12 hours post co-incubation of SARS-CoV-2 and CoVR-MV. 1, SARS-CoV-2; 2, SARS-CoV-2+CoVR-MV.

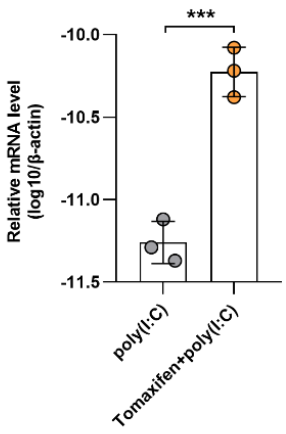

Figure S15. Relative mRNA levels of *IFN-β* in macrophages after co-incubation with poly(I:C) and Tomaxifen for 12 h (n=3). Macrophages were treated with Tamoxifen (10 mM) for 2 hours followed by 1μg/mL of poly(I:C) stimulation for 12 hours to analyze *IFN-β* mRNA expression. Data were shown as mean ± SD. Statistical analyses were performed using unpaired *t*-test. p-values <0.05 was considered significant: ***P <0.001.

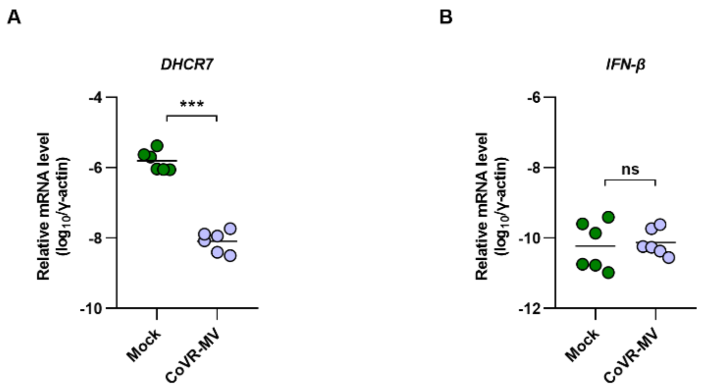

Figure S16. Relative mRNA levels of (A) *DHCR7* and (B) *IFN-β* in macrophages after co-incubation for 12 h (n=6). Data were shown as mean ± SD. Statistical analyses were performed using unpaired *t*-test. p-values <0.05 was considered significant: ***P <0.001, ns indicated no significance to the positive control (p>0.05).

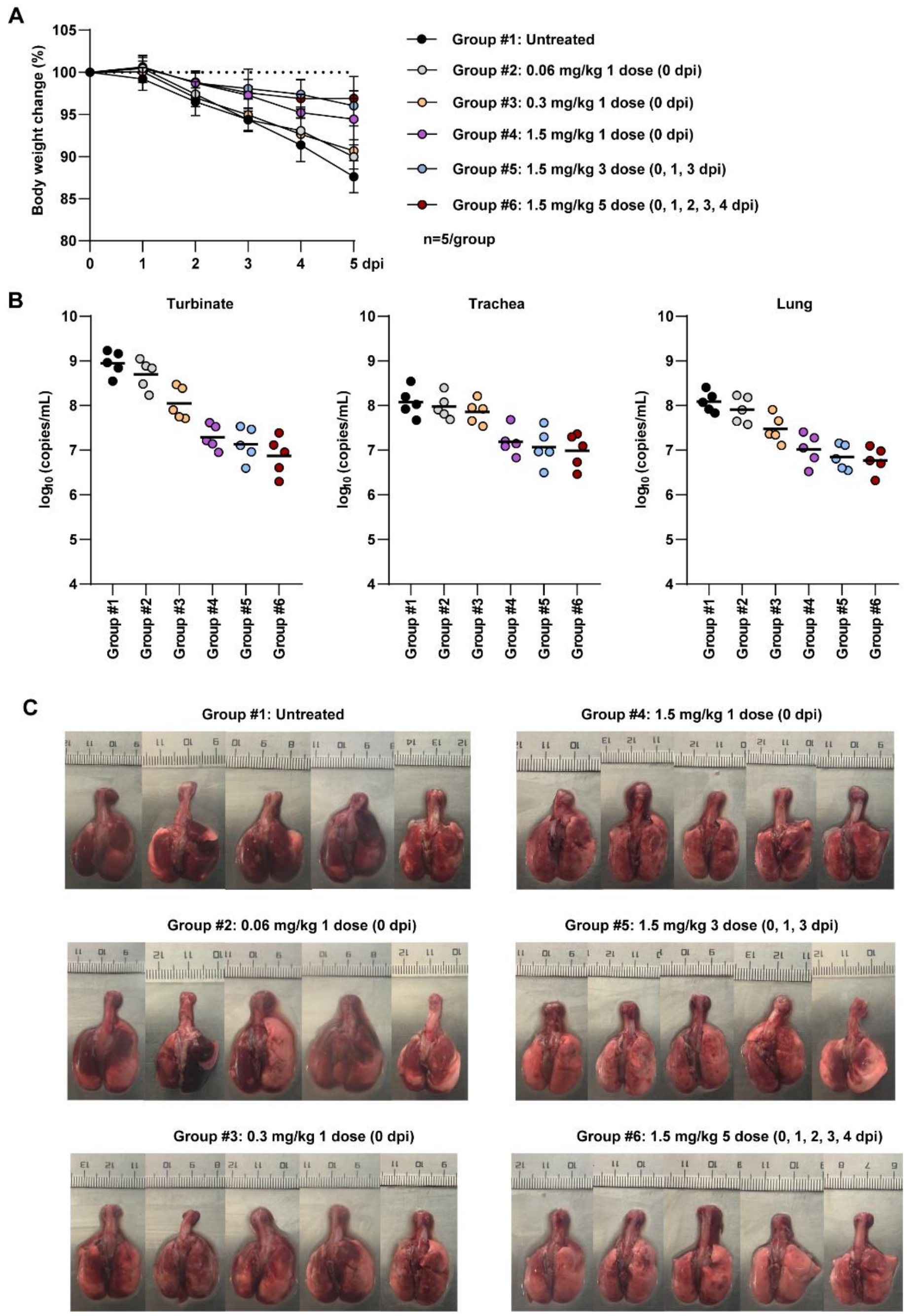

Figure S17. Theraputeic efficency of different doses and regimens of CoVR-MV in SARS-CoV-2 infected hamsters. After intranasal inoculation of 1×10^4 PFU of prototype SARS-CoV-2, hamsters were treated with different doses and regimens of CoVR-MV: 0.06 mg/kg one dose at 0 dpi (Group #2); 0.3 mg/kg one dose at 0 dpi (Group #3); 1.5 mg/kg one dose at 0 dpi (Group #4); 1.5 mg/kg three dose at 0, 1, 3 dpi (Group #5); 1.5 mg/kg five dose at 0, 1, 2, 3, 4 dpi (Group #6). The SARS-CoV-2 infected hamsters without reatment were set as controls ((Group #1). All of the hamsters survived at 5 dpi. A) Body weight changes of the survived hamsters from 0

to 5 dpi were recorded (n=5). B) Viral RNA levels in turbinate, trachea and lung tissues collected at 5 dpi were measured by RT-PCR (n=5). The primers of SARS-CoV-2 ORF1ab gene were used. C) Representative gross images for lung lobes collected at 5 dpi were shown (n=5).

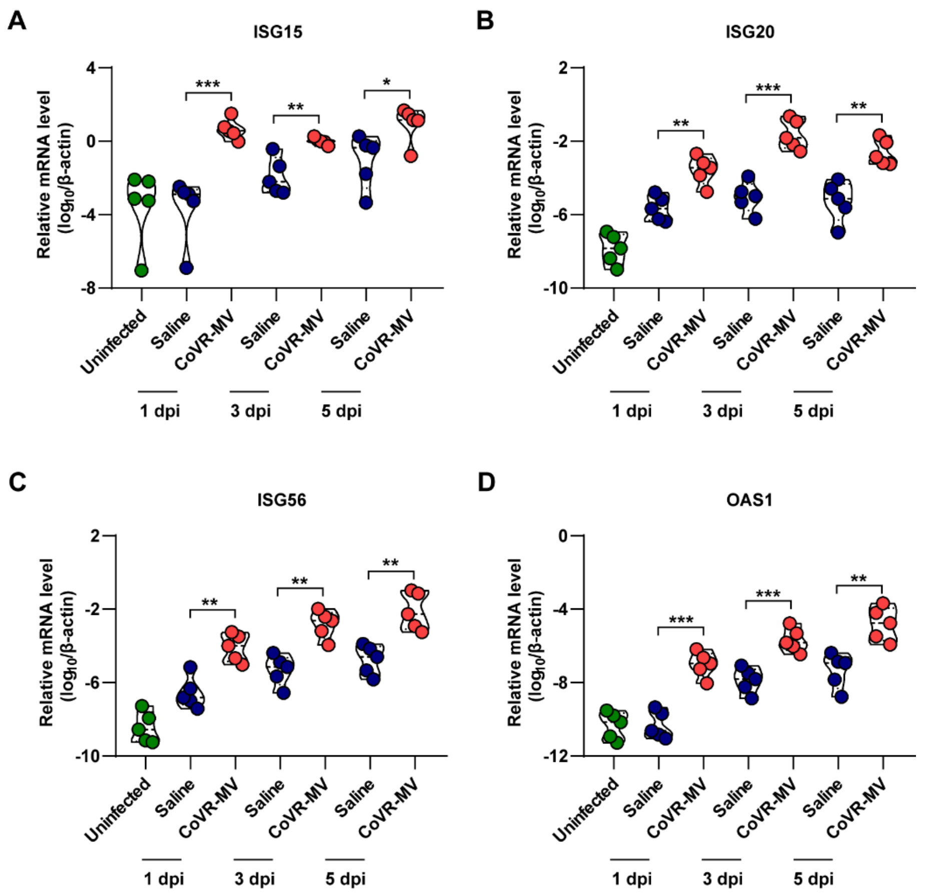

Figure S18. Relative mRNA levels of (A) ISG15, (B) ISG20, (C) ISG56 and (D) OAS1 in the lung tissues collected from euthanized hamsters at 1, 3 and 5 dpi, respectively (n=5). The mRNA levels were standardized to the house-keeping gene γ-actin.

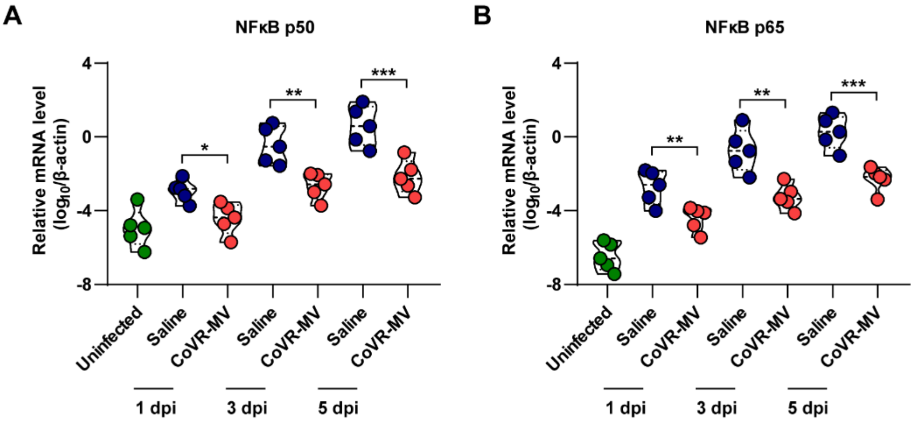

Figure S19. Relative mRNA levels of (A) NFκB p50 and (B) NFκB p65 in the lung tissues collected from euthanized hamsters at 1, 3 and 5 dpi, respectively (n=5). The mRNA levels were standardized to the house-keeping gene γ-actin.

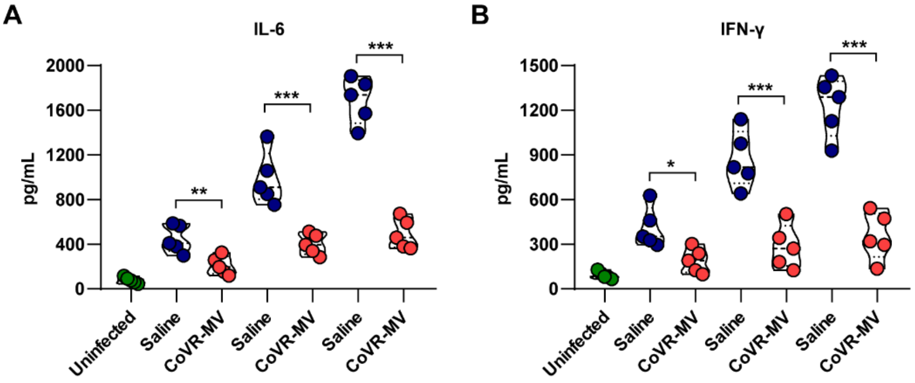

Figure S20. Concentration of (A) IL-6 and (B) IFN-γ in the lung tissues collected from euthanized hamsters at 1, 3 and 5 dpi, respectively (n=5).

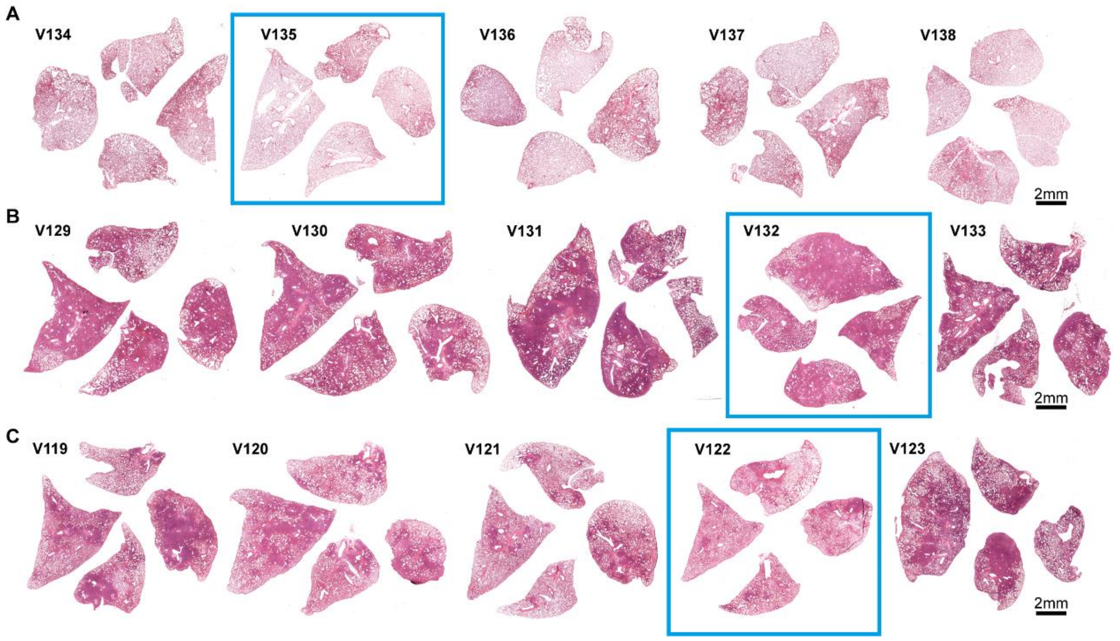

Figure S21. H&E staining of lung lobes collected from hamsters infected with SARS-CoV-2 prototype strain. All of the hamsters were sacrificed at 5 dpi. For each hamster, four lung lobes were fixed in formalin for pathological analysis. H&E staining for lung lobe sections collected from SARS-CoV-2 prototype strain infected hamsters (C) with or (B) without one-dose administration of CoVR-MV were screened by an auto-microscope system. A) The lung lobe sections collected from hamsters without infection were set as a mock group. The representative images in the blue box were shown in Figure 5.

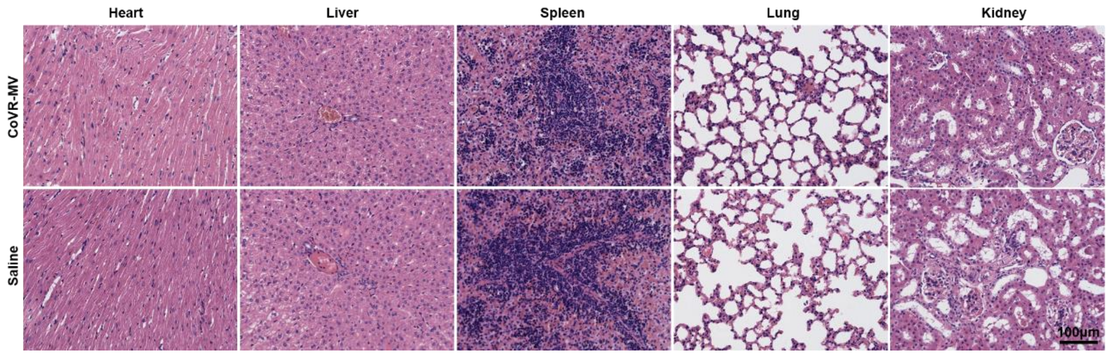

Figure S22. H&E staining of major organs collected from hamsters treated with sequential five-dose of CoVR-MV and Saline (one dose per day).

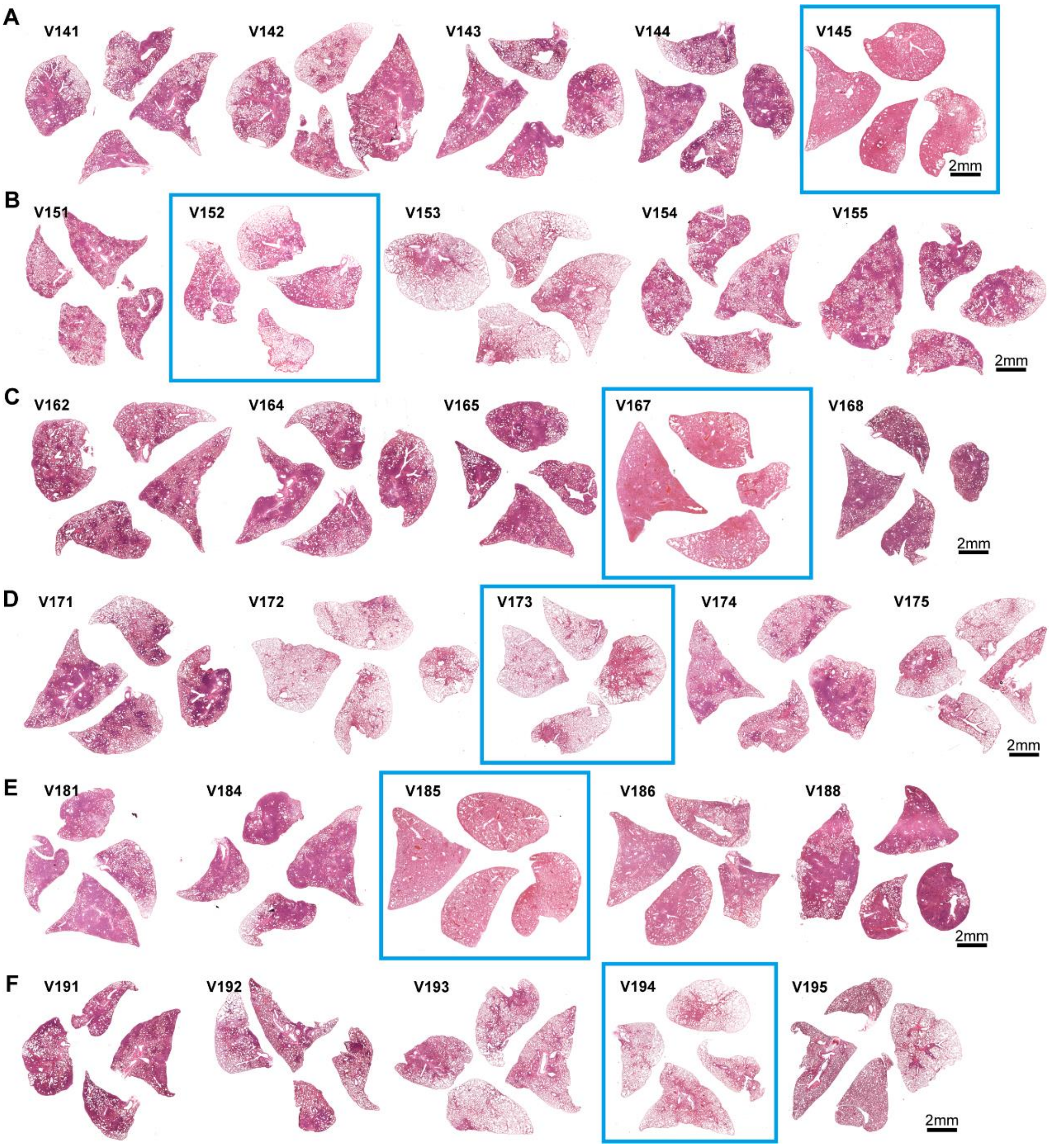

Figure S23. H&E staining of lung lobes collected from hamsters infected with SARS-CoV-2 prototype virus, 614G strain and B.1.351 stain. All of the hamsters were sacrificed at 5 dpi. For each hamster, four lung lobes were fixed in formalin for pathological analysis. H&E staining for lung lobe sections collected from SARS-CoV-2 prototype virus infected hamsters (B) with or (A) without therapy of CoVR-MV. H&E staining for lung lobe sections collected from SARS-CoV-2 614G strain infected hamsters (D) with or (C) without therapy of CoVR-MV. H&E staining for lung lobe sections collected from SARS-CoV-2 B.1.351 strain infected hamsters (F) with or (E) without therapy of CoVR-MV. The H&E staining results were screened by an auto-microscope system. The representative images in the blue box were shown in Figure 6.

Table S1. Detail Pathological Scores for H&E Staining of Hamster Lung Lobes in Figure S21.

Group	Identifier	Pathological lesions: Alveolar septum hyperplasia and consolidation	Pathological lesions: Pulmonary edema, hemorrhage and mucus suppository	Pathological lesions: Recruitment and infiltration of inflammatory cells	Comprehensive pathological score
Uninfected	V134	1+1+1+1	1+1+0+0	0+0+0+0	2+2+1+1
	V135	1+0+0+0	1+1+0+0	0+0+0+0	2+1+0+0
	V136	1+1+0+0	1+2+0+0	0+0+0+0	2+3+0+0
	V137	1+1+0+0	2+2+1+0	0+0+0+0	3+3+1+0
	V138	1+0+0+0	2+0+0+0	0+0+0+0	3+0+0+0
Saline	V129	3+3+2+1	4+4+3+2	4+4+3+1	11+11+8+4
	V130	3+3+2+2	4+2+2+2	4+3+3+3	11+7+7+7
	V131	4+3+1+1	4+4+2+1	4+4+2+1	12+11+5+3
	V132	4+3+3+2	4+3+3+2	4+4+3+2	12+10+9+6
	V133	3+3+2+2	3+3+3+2	3+3+2+2	9+9+7+6
CoVR-MV	V119	3+1+1+0	3+2+1+0	3+2+1+1	9+5+3+1
	V120	2+1+1+0	2+2+2+1	3+2+2+1	7+5+5+2
	V121	1+1+1+1	1+1+1+1	2+1+1+0	4+3+3+2
	V122	1+1+1+1	1+1+1+1	1+1+0+0	3+3+2+2
	V123	3+2+1+0	3+2+1+1	3+2+2+0	9+6+4+1

Table S2. Detail Pathological Scores for H&E Staining of Hamster Lung Lobes in Figure S23.

Group	Identifier	Pathological lesions: Alveolar septum hyperplasia and consolidation	Pathological lesions: Pulmonary edema, hemorrhage and mucus suppository	Pathological lesions: Recruitment and infiltration of inflammatory cells	Comprehensive pathological score
PT	V141	3+3+2+1	3+3+3+2	3+3+3+2	9+9+8+5
	V142	3+2+2+1	4+3+3+1	4+3+2+1	11+8+7+3
	V143	3+3+2+1	3+3+3+1	3+3+3+1	9+9+8+3
	V144	4+3+2+2	4+3+2+2	4+4+2+2	12+10+6+6
	V145	4+4+3+3	4+4+3+3	4+3+3+3	12+11+9+9
PT +CoVR-MV	V151	2+2+1+1	2+2+1+1	3+2+1+0	7+6+3+2
	V152	2+2+1+0	2+2+1+0	1+1+0+0	5+5+2+0
	V153	1+1+1+1	1+1+1+1	1+1+1+1	3+3+3+3
	V154	1+1+1+1	2+2+1+1	2+2+1+1	5+5+3+3
	V155	2+2+2+1	2+2+2+1	2+2+2+1	6+6+6+3
614G	V162	3+3+3+2	3+3+2+2	3+3+2+2	9+9+8+6
	V164	3+3+3+1	3+3+3+2	3+3+3+1	9+9+9+4
	V165	3+3+3+2	3+3+3+2	4+4+3+2	10+10+9+6
	V167	4+4+3+3	4+4+3+3	4+4+2+2	12+12+8+8
	V168	3+3+3+3	3+3+3+3	4+4+3+3	10+10+9+9
614G +CoVR-MV	V171	2+2+1+1	2+2+1+1	3+3+1+1	7+7+3+3
	V172	0+0+0+0	1+1+1+1	0+0+0+0	1+1+1+1
	V173	2+1+0+0	2+1+0+0	1+0+0+0	5+2+0+0
	V174	2+1+0+0	2+1+1+0	3+1+0+1	7+3+1+1
	V175	1+1+0+0	1+1+1+0	1+0+0+0	3+2+1+0
B.1.351	V181	4+3+3+1	4+3+3+1	4+4+3+2	12+10+9+4

	**V184**	4+3+3+3	4+4+3+2	4+4+3+3	12+11+9+8
	**V185**	4+4+4+4	4+4+4+4	4+4+3+3	12+12+11+11
	**V186**	3+3+3+2	4+4+3+2	4+4+3+2	11+11+9+6
	**V188**	4+4+3+3	4+4+3+3	4+4+4+3	12+12+10+10
**B.1.351+ CoVR-MV**	**V191**	2+2+2+1	2+2+2+2	2+2+2+2	6+6+6+5
	**V192**	1+1+0+0	1+1+1+1	1+1+1+1	3+3+2+2
	**V193**	2+1+1+0	2+1+1+0	2+1+0+0	6+3+2+0
	**V194**	1+1+0+0	2+2+1+1	0+0+0+0	3+3+1+1
	**V195**	1+1+1+0	2+2+2+1	0+0+0+0	3+3+3+1

Table S3. The gene-specific primers (5’ to 3’) used for RT-PCR of cytokine profiling

**Genes**	**Forward**	**Reverse**
**Hamster IFN-γ**	TGTTGCTCTGCCTCACTCAGG	AAGACGAGGTCCCCTCCATTC
**Hamster IL-6**	AGACAAAGCCAGAGTCATT	TCGGTATGCTAAGGCACAG
**Hamster TNF-α**	TGAGCCATCGTGCCAATG	AGCCCGTCTGCTGGTATCAC
**Hamster IFN-α**	CTGGTGGCTGTGAGGAAATA	AGCAAGTTGGCTGAGGAAGA
**Hamster IFN-β**	TTGTGCTTCTCCACTACAGC	GTGTCTAGATCTGACAACCT
**Hamster ISG15**	AAAGCCTACAGCCATGACCT	TTAGTCAGGGGCACCAGGAA
**Hamster ISG20**	ACGGATTACAGAACCCGAGTC	CTGCAGGATCTCTAGTCTGGCTT
**Hamster ISG56**	ACTCTGTGAAGTCTAGGGACAGG	TCATCCGCGACAAGACGATG
**Hamster MX1**	GCGCTTCCAGACTCTTCTGA	CCTAAGATACATGCGATGGCG
**Hamster OAS1**	AGCTCCTCTGATCTCCAGCA	TGTTGACATCACCCAGGACA
**NFkB p65**	AGCAGGAGCAAACTATCGCC	AAAGAGCGAAGTACCCGGTTG
**NFkB p50**	TTATCTCGCAGCCCATCCAC	TGCCATCCGTTCTCGTCATC
**Hamster γ-actin**	ACAGAGAGAAGATGACGCAGATAATG	GCCTGAATGGCCACGTACA
